# The *Vibrio cholerae* RND efflux systems impact virulence factor production and adaptive responses via periplasmic sensor proteins

**DOI:** 10.1371/journal.ppat.1006804

**Published:** 2018-01-05

**Authors:** X. Renee Bina, Mondraya F. Howard, Dawn L. Taylor-Mulneix, Vanessa M. Ante, Dillon E. Kunkle, James E. Bina

**Affiliations:** Department of Microbiology and Molecular Genetics, University of Pittsburgh School of Medicine, Pittsburgh, PA, United States of America; Stanford University School of Medicine, UNITED STATES

## Abstract

Resistance-nodulation-division (RND) efflux systems are ubiquitous transporters in Gram-negative bacteria that are essential for antibiotic resistance. The RND efflux systems also contribute to diverse phenotypes independent of antimicrobial resistance, but the mechanism by which they affect most of these phenotypes is unclear. This is the case in *Vibrio cholerae* where the RND systems function in antimicrobial resistance and virulence factor production. Herein, we investigated the linkage between RND efflux and *V*. *cholerae* virulence. RNA sequencing revealed that the loss of RND efflux affected the activation state of periplasmic sensing systems including the virulence regulator ToxR. Activation of ToxR in an RND null mutant resulted in ToxR-dependent transcription of the LysR-family regulator *leuO*. Increased *leuO* transcription resulted in the repression of the ToxR virulence regulon and attenuated virulence factor production. Consistent with this, *leuO* deletion restored virulence factor production in an RND-null mutant, but not its ability to colonize infant mice; suggesting that RND efflux was epistatic to virulence factor production for colonization. The periplasmic sensing domain of ToxR was required for the induction of *leuO* transcription in the RND null mutant, suggesting that ToxR responded to metabolites that accumulated in the periplasm. Our results suggest that ToxR represses virulence factor production in response to metabolites that are normally effluxed from the cell by the RND transporters. We propose that impaired RND efflux results in periplasmic metabolite accumulation, which then activates periplasmic sensors including ToxR and two-component regulatory systems to initiate the expression of adaptive responses.

## Author summary

Multidrug efflux systems belonging to the RND superfamily contribute to the expression of diverse phenotypes in Gram-negative bacteria, but the mechanisms linking RND efflux to these phenotypes is unclear. Herein, we provide evidence suggesting that the *V*. *cholerae* RND systems influence global transcription patterns by extruding cell metabolites. Inhibition of RND efflux causes cell metabolites to accumulate intracellularly where they stimulate periplasmic sensors including the virulence regulator ToxR. The sensor proteins then initiate the expression of transcriptional responses, which in *V*. *cholerae* includes ToxR-mediated repression of virulence factor production. This study sheds light on the native functions of RND systems in Gram-negative bacteria and suggests a new paradigm for RND-mediated efflux in environmental sensing and adaptation.

## Introduction

Antimicrobial resistance is an expanding global health threat. A ubiquitous mechanism in Gram-negative bacteria that contributes to drug resistance is the ability to reduce antimicrobial uptake. Reduced uptake involves decreasing the rate of antimicrobial diffusion across the outer membrane (OM) combined with the expression of efflux systems (reviewed in [1]). Reduced OM permeability is most often mediated by lipid A modification and/or altered porin production. Efflux systems function synergistically with reduced OM permeability to export antimicrobial compounds that have crossed the OM. The Resistance-Nodulation-Division (RND) efflux systems play a predominant role in this process because they often exhibit broad substrate specificity that provides cross-resistance to multiple classes of antimicrobials.

The RND efflux systems are ubiquitous in Gram-negative bacteria. They consist of an inner membrane pump protein, a periplasmic membrane fusion protein, and an outer membrane pore protein [1]. These three components function together to efflux substrates from the cytoplasm and periplasm to the external environment. Although many RND systems are linked to antimicrobial resistance, recent studies have implicated them in diverse phenotypes including metabolism, biofilm production, iron acquisition, and virulence [2–4]. These latter observations suggest that individual RND transporters fulfill specific phenotypes in the cell, but the mechanisms by which they contribute to these phenotypes is poorly understood.

*Vibrio cholerae* is a Gram-negative bacterium that causes cholera; an acute diarrheal disease affecting ~3 million people per year [5]. To cause disease, *V*. *cholerae* must adapt to the host gastrointestinal tract. This includes expressing genes that allow it to resist host antimicrobials and to produce virulence factors that facilitate colonization. This process is mediated in part by the membrane associated transcription factor ToxR. ToxR is a global regulator that regulates antimicrobial resistance and virulence genes in response to environmental cues (reviewed in [6]). ToxR is an essential member of the ToxR virulence regulon where it functions with TcpP to activate the expression of genes encoding for the virulence factors cholera toxin (CT) and the toxin coregulated pilus (TCP). Independent of TcpP, ToxR contributes to antimicrobial resistance by regulating porin production and lipid A remodeling [7, 8]. The *in vivo* cues that modulate ToxR activity are poorly understood, but studies suggest that the ToxR periplasmic domain (PPD) serves as a sensor to transduce environmental cues to alter the activity of its DNA binding domain [9–12].

*V*. *cholerae* encodes six RND efflux systems that share TolC as their OM pore [13, 14]. Mutants lacking RND transporters, or wild type (WT) treated with RND efflux inhibitors, are hypersensitive to antibiotics, bile salts, fatty acids, and cationic antimicrobial peptides (CAPs) [14, 15]. In addition, the *V*. *cholerae* RND efflux systems are required for CT and TCP production and colonization of the infant mouse intestine [14]. The mechanism linking RND efflux to CT/TCP production is unknown, but was correlated with reduced *tcpP* transcription [14]. Collectively these results indicated that the RND transporters have pleiotropic effects on *V*. *cholerae* pathogenesis.

Herein, we investigated the function of the RND transporters in *V*. *cholerae* virulence. We document that RND efflux had wide-ranging effects on the *V*. *cholerae* transcriptome, suggesting that they have critical functions in cell physiology. In the absence of RND efflux, cellular metabolites that are normally effluxed by the RND transporters appeared to accumulate in the periplasm where they affected the activation state of periplasmic sensing proteins like ToxR and two-component systems. We further show that RND efflux plays a dual role in pathogenesis, being required for the expression of virulence genes and for resistance to antimicrobial compounds that are present in the host. Altogether our results suggest that RND efflux can influence the expression of adaptive responses by modulating the intracellular concentration of cell metabolites.

## Results

### RND efflux regulates *leuO* transcription

The loss of RND efflux in *V*. *cholerae* resulted in attenuated CT and TCP production [14]. To identify genes involved in this process, we determined the transcriptome of RND efflux negative *V*. *cholerae* strain JB485 by RNA sequencing (RNAseq). Total RNA was isolated and sequenced from JB485 and WT following growth under virulence inducing conditions (AKI conditions). The data from three independent experiments was then analyzed as described in the methods to identify differentially expressed genes. The RNAseq identified 373 genes that were differentially expressed in JB485 (S1 Table). The largest category of differentially expressed genes were those of unknown function (132 genes) followed by metabolism (60 genes), transport and binding (75 genes) and regulatory genes (30 genes) (Table 1). Consistent with previous studies, the expression of many virulence genes was reduced in JB485 (S1 Table). This included genes involved in the production of CT, TCP and multifunctional-autoprocessing repeats-in-toxin (MARTX). Surprisingly, the operons encoding for the VexAB, VexCD, VexGH, VexIJK and VexLM RND efflux systems were upregulated. Additionally, many porin genes were also differentially expressed including the upregulation of VC0972, *ompK*, *ompW* and downregulation of *ompV* and *ompS*. As reduced OM permeability functions synergistically with active efflux to effect high-level antimicrobial resistance [1], it is possible that the altered expression of the RND systems and porins reflects adaptive responses to modulate the OM barrier properties in response to the loss of RND efflux. Altogether these results suggest that impaired RND efflux activates global responses that facilitate the expression genes involved in environmental adaptation.

**Table 1:** Categories of differentially expressed genes in JB485.

Functional group:		Upregulated genes:	Downregulated genes:	Total:	Percent:
	Amino acid biosynthesis	9	1	10	2.7
	Biosynthesis of cofactors, prosthetic groups, and carriers	2	1	3	0.8
	Cell envelope	8	3	11	2.9
	Cellular processes	8	1	9	2.4
	Metabolism	39	21	60	16.1
	Conserved, hypothetical, and unknown	74	57	131	35.4
	DNA replication, recombination, and repair	3	0	3	0.8
	Other categories	0	2	2	0.5
	Pathogenesis	3	16	19	5.1
	Protein fate	16	3	19	5.1
	Regulatory functions	21	9	30	8.0
	Transport and binding proteins	27	48	75	20.1
	**TOTAL:**	**210**	**163**	**372**	**100**

We hypothesized that the link between RND efflux and virulence factor production was being mediated by a transcription factor. There were 30 differentially expressed regulatory genes identified in the RNAseq results (S1 Table); 21 of which were upregulated. Two-component signal transduction regulatory systems (TCS) made up 57% of these identified regulatory genes, which is highly enriched compared to TCSs represented in the *V*. *cholerae* genome (~17%). TCS consist of a membrane sensor that relays environmental signals to a response regulator to induce a cellular response. As such, the enrichment of TCSs among the identified regulatory genes suggests that the loss of RND efflux may have resulted in physiological alterations in the periplasmic compartment; perhaps due to the accumulation of substrates that are normally effluxed from the periplasm by the RND systems. Several of the identified TCSs contribute to environmental adaptation and pathogenesis including CpxRA [16–18], CarRS [19, 20], VieSAB [21], and OmpR [22]. We excluded several of the identified genes as virulence regulators in JB485 including *cpxRA*, *vexR*, and *breR* as previous studies suggested that they did not affect CT and TCP production [18, 23, 24]. Among the remaining regulatory genes, the LysR-family transcription factor *leuO* was one of the most highly upregulated (~6-fold) in JB485. LeuO is a global regulator that has been linked to multiple *V*. *cholerae* phenotypes including ToxR regulon expression [11, 12, 25–27]. We therefore further investigated *leuO* in JB485.

**Fig 1:**
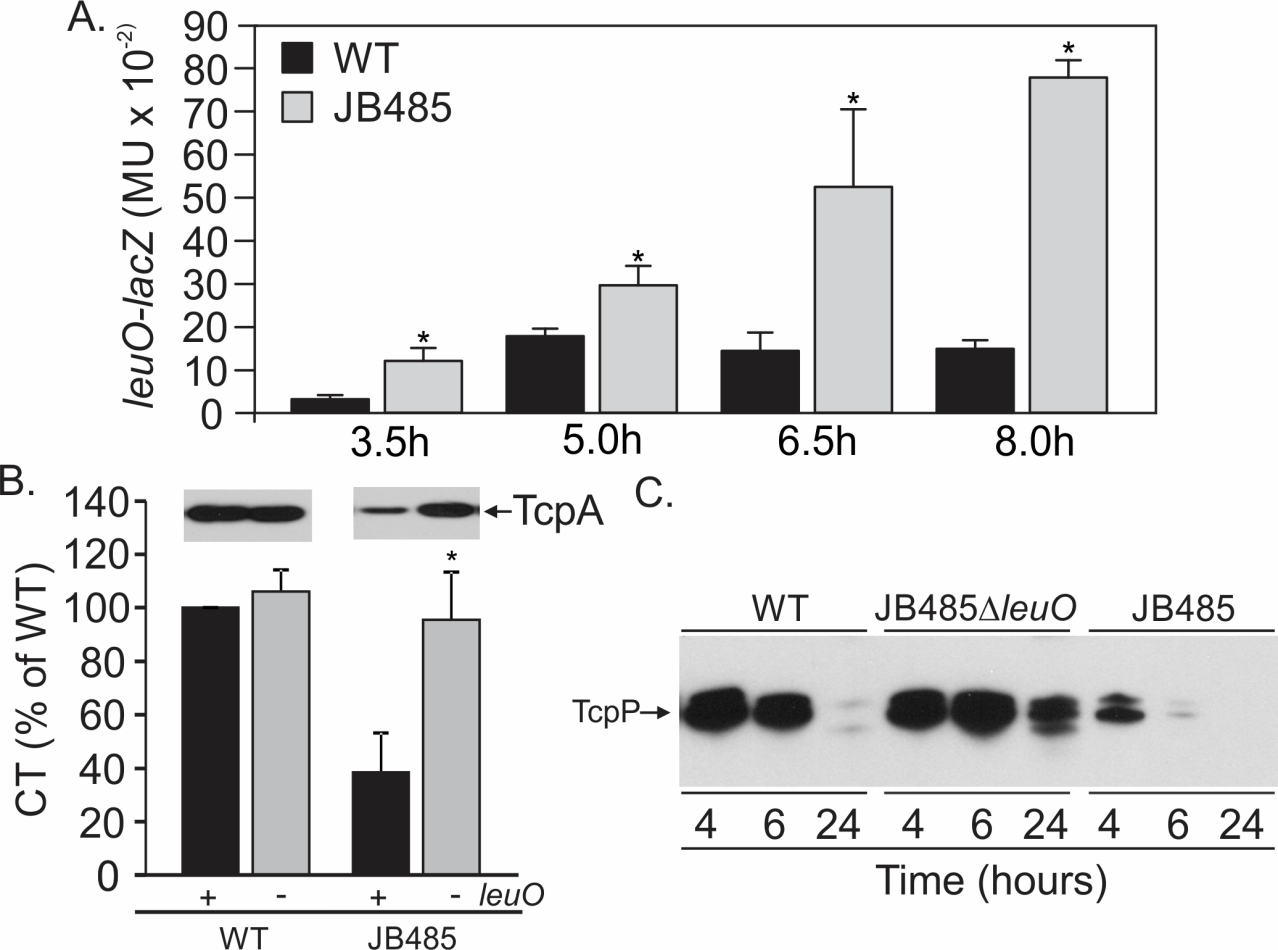
LeuO represses virulence in RND-deficient *V*. *cholerae*. *V*. *cholerae* were cultured under AKI conditions and processed as described in the methods. (A) *leuO* expression in WT and JB485 bearing pXB266 (*leuO-lacZ*). (B) Deletion of *leuO* restores CT and TcpA production (inset) in JB485. (C) TcpP Western blot showing that *leuO* deletion restores TcpP production in JB485. The data presented (A & B) is the means ± SD from at least three independent experiments. *, P≤0.05.

To confirm that *leuO* expression was increased in JB485 we introduced a *leuO-lacZ* reporter into WT and JB485 and quantified *leuO* expression over time during growth under AKI conditions. The results showed increased *leuO* expression in JB485 relative to WT at each time point (Fig 1A). *leuO* expression was ~4-fold higher in JB485 than WT at 3.5h and its expression continued to increase through the duration of the experiment, confirming the RNAseq results.

Previous studies showed differential contributions of the six *V*. *cholerae* RND transporters to antimicrobial resistance and virulence factor production [28, 29]. VexB, VexD, VexH, and VexK pumps functioned in in vitro antimicrobial resistance while all six pumps contributed to CT and TCP production. Given this, we tested if individual RND pumps differentially affected *leuO* expression by quantifying *leuO*-*lacZ* expression in a panel of RND mutant strains following growth under AKI conditions. The results showed that cells lacking the *vexB*, *vexD* or *vexBD* RND pumps did not affect *leuO* expression (S1 Fig). The expression of *leuO-lacZ* significantly increased in the Δ*vexBDH* mutant. Deletion of four RND pumps (Δ*vexBDHK*) increased *leuO* expression to a level similar to JB485 (Δ*vexBDFHKM*). This indicated that *vexBDHK* were linked to the increased *leuO* transcription. The expression of *leuO* was unaffected in strains DT422 (Δ*vexBFHKM; vexD*+) and JB464 (Δ*vexDFHKM*, *vexB*+), suggesting that the presence of *vexB* or *vexD* alone could suppress the RND efflux-dependent induction of *leuO* transcription. The fact that *vexB* or *vexD* suppressed *leuO* expression is consistent with previous studies showing redundancy among the RND efflux systems [14, 28, 29].

### Mutation of *leuO* restores virulence factor production in an RND negative mutant

Increased *leuO* expression in the RND negative mutant JB485 suggested that LeuO repressed CT and TCP production. To test this, we compared CT and TcpA production in WT, JB485, Δ*leuO* and JB485Δ*leuO* following growth under AKI conditions. Consistent with previous results [14], CT and TcpA production were attenuated in JB485 relative to WT (Fig 1B). Deletion of *leuO* in WT did not significantly affect CT or TcpA production. By contrast, *leuO* deletion in JB485 restored CT and TcpA production to WT levels (Fig 1B), confirming that increased *leuO* expression was responsible for reduced CT and TcpA production in JB485.

Transcription of *tcpPH* was reported to be repressed in JB485 [14]. We therefore tested if *leuO* deletion in JB485 affected TcpP production under AKI conditions. As shown in Fig 1C, TcpP production in WT peaked at 4h and then declined to very low levels following overnight growth. TcpP production in JB485 was greatly reduced relative to WT, and still declined over time. Deletion of *leuO* in JB485 elevated TcpP production, with TcpP peaking at 6h before declining. In contrast to WT, TcpP was still relatively abundant in overnight JB485Δ*leuO* cultures (Fig 1C). Although TcpP production appeared to be higher in JB485Δ*leuO* at 6h and 24h, CT and TcpA production in JB485Δ*leuO* was like WT (Fig 1B); this is likely due to TcpP indirectly regulating CT and TCP production. Collectively these results suggested that the loss of RND efflux increased *leuO* expression and that LeuO was responsible for reduced TcpP, CT and TcpA production.

### LeuO represses the ToxR regulon in the absence of RND efflux

**Fig 2:**
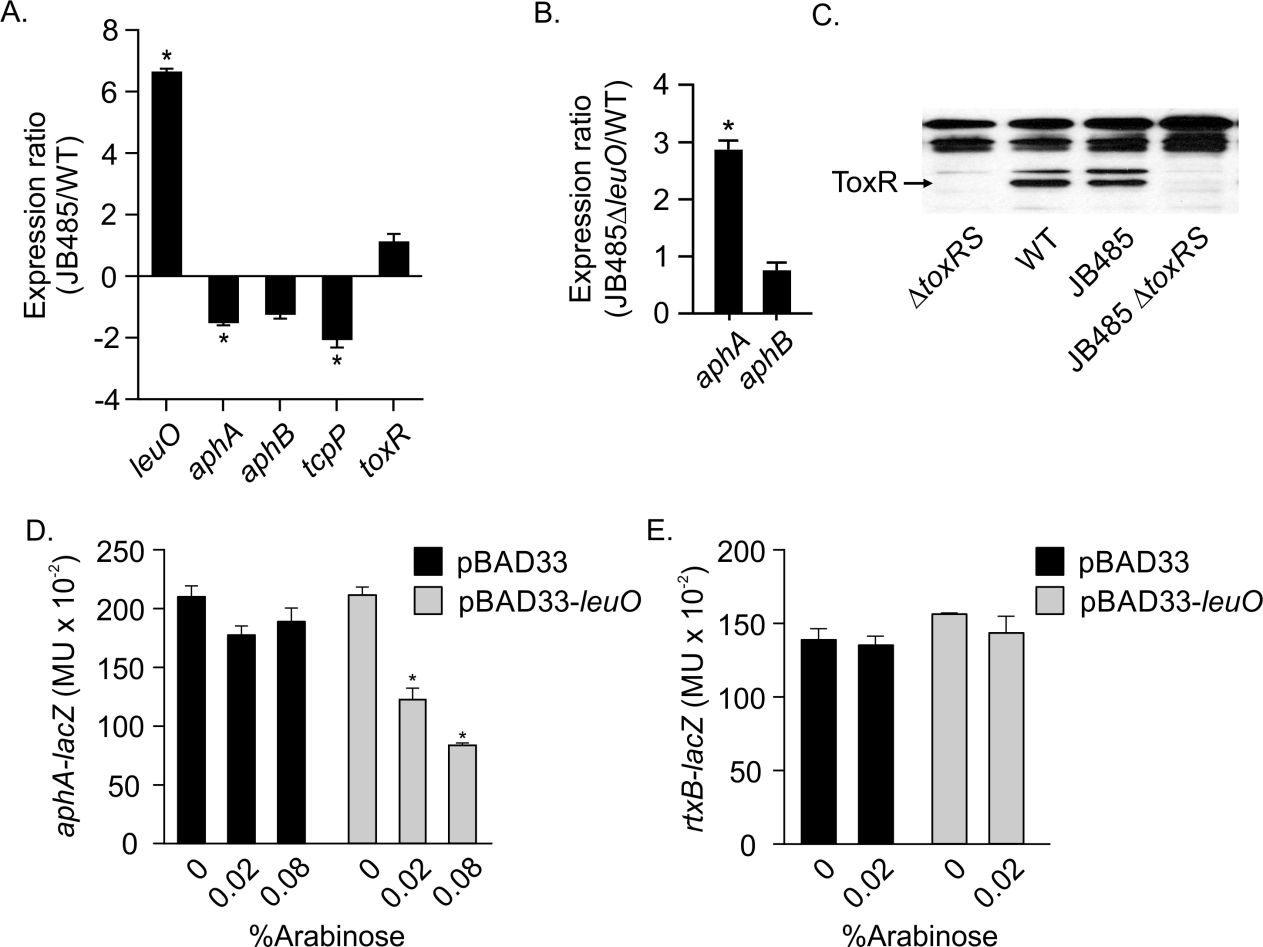
LeuO represses *aphA* in JB485. (A & B) The indicated *V*. *cholerae* strains were cultured under AKI conditions for 3.5h when total RNA was isolated and used for qRT-PCR to quantify *aphA*, *aphB*, *tcpP* and *toxR* expression. P-values are relative to a hypothetical ratio of 1.0 and were determined using the Student’s *t*-test. * P<0.05. (C) ToxR Western blot. The indicated strains were grown under AKI conditions for 6h when aliquots were collected, normalized by OD_600_, and subjected to Western blotting with anti-ToxR antibody. The extra non-specific bands serve as loading controls. (D & E) Overexpression of *leuO* represses *aphA-lacZ*, but not *rxtB-lacZ* expression in *E*. *coli*. *E*. *coli* containing pBAD33::*leuO* or pBAD33 and a (D) *aphA-lacZ* or (E) *rtxB-lacZ* reporter plasmid was cultured in LB broth to mid-log phase (5h) when β-galactosidase activity was quantified. The data are the mean ±SD of 3 independent experiments. * P≤0.05 relative to the no arabinose control.

We hypothesized that if *leuO* was repressing specific genes in the ToxR regulon, then ectopic expression of the repressed genes would restore CT production. We tested this by overexpressing *aphA*, *aphB*, *tcpPH* and *toxT* in JB485 and quantifying CT production following growth under AKI conditions. Both *toxT* and *tcpPH* restored CT production (S2A Fig). As *toxT* is downstream of *tcpPH* in the ToxR regulon, this is consistent with previous studies linking *tcpPH* repression to attenuated CT/TCP production in JB485. AphA and AphB function upstream of *tcpPH* and bind to the *tcpPH* promoter to activate its expression [30]. The expression of *aphA* restored CT production in an arabinose-concentration dependent manner (S2B Fig). The expression of *aphB* partially restored CT production in JB485, but in contrast to *aphA*, CT production was independent of the arabinose concentration (S2B Fig). This suggested that decreased *tcpPH* expression may be due to LeuO repressing *aphA* transcription. We further tested this by using qRT-PCR to quantify *aphA*, *aphB*, *leuO*, and *tcpP* expression in WT and JB485 during growth under AKI conditions. The results showed ~6-fold increase in *leuO* expression and a ~2-fold decrease in *tcpP* expression in JB485 relative to WT (Fig 2A), while *aphA* expression was reduced by ~1.5-fold. By contrast, the expression of *aphB* and *toxR* was not significantly changed. Consistent with the qRT-PCR results we also observed that ToxR and AphB protein production was not different between WT and JB485 (Fig 2C and S3 Fig). These results suggested that *leuO* repressed *aphA* expression in JB485.

To further confirm that LeuO repressed *aphA* transcription in JB485, we compared *aphA* expression in JB485Δ*leuO* to WT. We hypothesized that if *leuO* was responsible for reduced *aphA* expression, then *aphA* expression should increase in JB485Δ*leuO*. The results showed an approximately 3-fold increase in *aphA* expression in JB485Δ*leuO* relative to WT, but no change in *aphB* expression, confirming our hypothesis (Fig 2B). Increased *aphA* transcription in JB485Δ*leuO* potentially explains the elevated TcpP protein observed in JB485Δ*leuO* (Fig 1C). These results provide additional evidence to suggest that LeuO-dependent repression of *aphA* decreased *tcpPH* expression and CT and TcpA production in JB485.

To address if LeuO was acting directly at the *aphA* promoter, we introduced pBAD33-*leuO* into *E*. *coli* bearing an *aphA-lacZ* reporter. Cultures were grown in the presence of arabinose to induce *leuO* expression before quantifying *aphA-lacZ* expression. The results showed an arabinose dose-dependent decrease in *aphA-lacZ* expression in the pBAD33-*leuO* culture and no effect on *aphA* expression in the pBAD33 control (Fig 2D). As an additional control we examined the effect of pBAD33-*leuO* on *rtxB*, which was also differentially regulated in JB485 (S1 Table). Overexpression of *leuO* did not affect *rtxB* expression in *E*. *coli*, indicating that LeuO did not directly regulate *rtxB* and exhibited specificity for the *aphA* promoter (Fig 2E). Gel-shift assays were also performed and showed that LeuO can directly bind to the *aphA* promoter (S4 Fig). Based on these results we concluded that LeuO directly repressed *aphA* transcription.

### ToxR positively regulates *leuO* in response to impaired RND efflux

**Fig 3:**
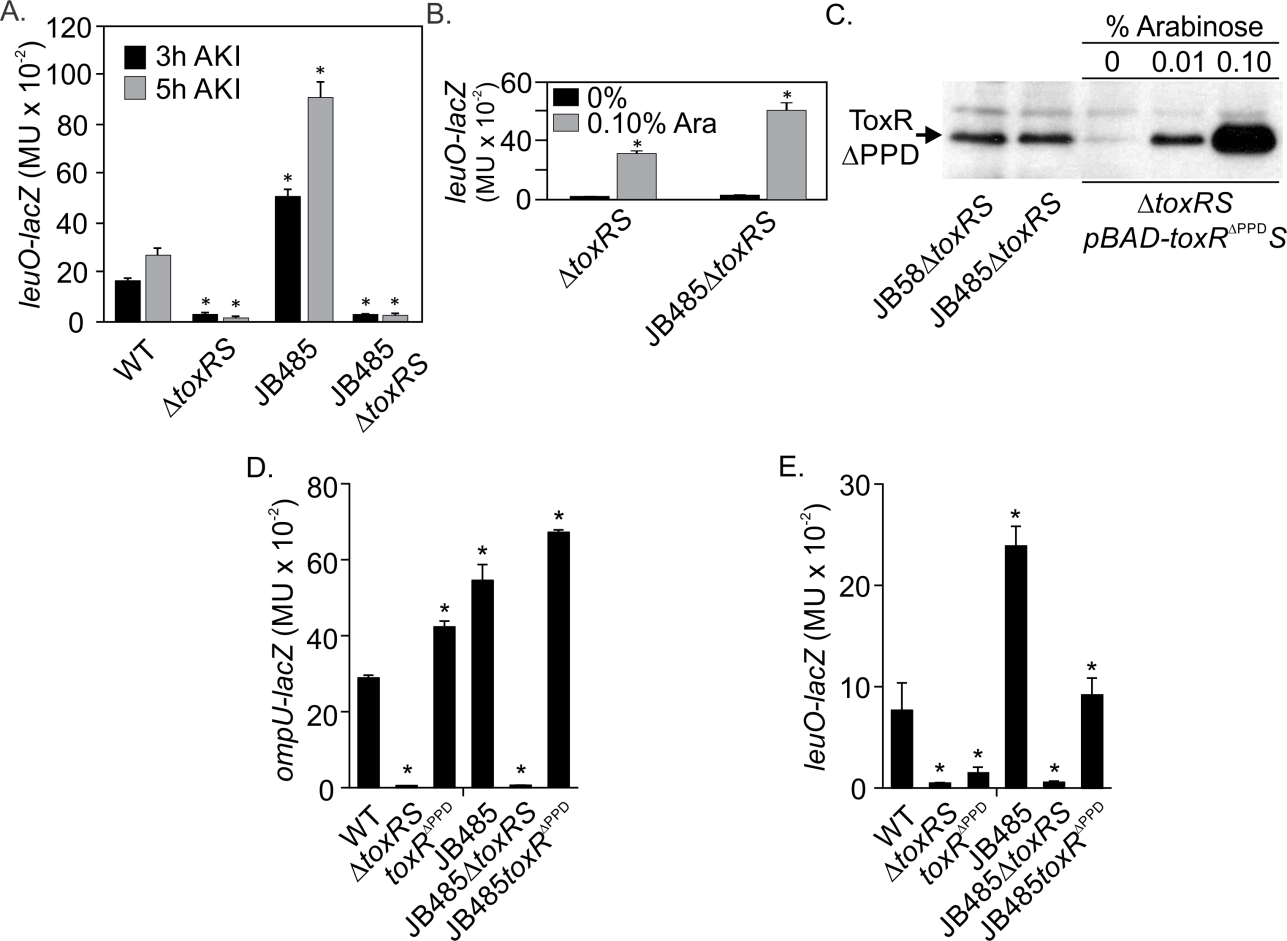
LeuO expression in JB485 is dependent upon the ToxR PPD. (A) Expression of *leuO* in JB485 requires *toxRS*. WT, Δ*toxRS* or JB485 bearing pXB266 *(leuO-lacZ)* were cultured under AKI conditions for 3h or 5h when β-galactosidase activity was quantified. (B) Ectopic *toxRS* expression complements for *leuO* expression. The Δ*toxRS* and JB485Δ*toxRS* mutants bearing pXB266 and pBAD33::*toxRS* were cultured under AKI conditions without or with 0.1% arabinose for 5h when β-galactosidase was quantified. (C) ToxR^ΔPPD^ production. Strains were grown under AKI conditions for 5h when aliquots were collected, normalized by OD_600_, and used for Western blotting with anti-ToxR antibody. A *toxRS* mutant carrying pBAD18::*toxR*^ΔPPD^*S* grown under AKI conditions in the presence of 0%, 0.01% and 0.1% arabinose is included as a marker for ToxR^ΔPPD^ protein. (D & E) Differential requirements of the ToxR PPD for *ompU* and *leuO* expression. The indicated strains carrying an *ompU-lacZ* or *leuO-lacZ* reporter were cultured under AKI conditions for 5h when β-galactosidase activity was quantified. *, P≤0.05 relative to the parental strain.

ToxR positively regulates *leuO* transcription in response to extracellular cues [11, 12]. We therefore tested if ToxR activated *leuO* transcription in cells lacking RND-mediated efflux. We cultured WT, Δ*toxRS*, JB485, and JB485Δ*toxRS* bearing pXB266 (*leuO-lacZ)* under AKI conditions and quantified *leuO* expression at 3h and 5h. The results showed increased *leuO* expression in JB485 by ~3.5-fold at 3h and 4.5-fold at 5h relative to WT (Fig 3A). By contrast, *leuO* expression was greatly diminished in JB485Δ*toxRS*, indicating that *toxRS* was required for *leuO* expression in the absence of RND efflux. To confirm that ToxR was responsible for *leuO* expression in JB485, we complemented *toxRS* mutants during growth under AKI conditions by introducing pBAD33::*toxRS* (pXB302) into the Δ*toxRS* and JB485Δ*toxRS* mutants bearing pXB266 (*leuO-lacZ*). Strains grown in the presence of arabinose increased *leuO* expression relative to the no arabinose control (Fig 3B). Interestingly, the magnitude of *leuO* expression in JB485Δ*toxRS* was ~30% greater than the efflux positive Δ*toxRS* mutant at the same arabinose concentration. While the significance of this is unclear, we speculate that it reflects differences in ToxR activation in the RND negative mutant vs WT background. These results support the conclusion that ToxR positively regulates *leuO* and that ToxR is responsible for increased *leuO* transcription in JB485. These results also suggest that RND mediated efflux may influence ToxR activation.

The ToxR PPD is thought to function as an environmental sensor and is required for the activation of *leuO* transcription in response to extracellular cues [11, 12]. Since *leuO* expression in JB485 was dependent on ToxR (Fig 3A), we tested if the ToxR PPD was required for *leuO* expression in the RND null mutant. Therefore, we generated chromosomal ToxRΔPPD mutants in WT and JB485 by truncating ToxR at amino acid 188 to remove the C-terminal PPD as previously reported (hereafter referred to as *toxR*^ΔPPD^) [31]. We then introduced pXB266 (*leuO-lacZ*) and pAL144 (*ompU-lacZ*) into WT, Δ*toxRS*, *toxR*^ΔPPD^, JB485, JB485Δ*toxRS* and JB485*toxR*^ΔPPD^, grew the resulting strains under AKI conditions, and quantified *leuO* and *ompU* expression.

We first validated that the *toxR*^ΔPPD^ allele was functional by examining *ompU* expression. Expression of *ompU* is dependent on ToxR, but independent of the ToxR PPD in rich media [31]. Consistent with this, *toxRS* deletion ablated *ompU* expression in WT and JB485 while PPD deletion had no effect on *ompU* expression (Fig 3D). In fact, *ompU* expression was elevated in the *toxR*^ΔPPD^ mutants relative to their WT parental strains; as previously observed [31]. These studies also revealed elevated *ompU* expression in JB485 relative to WT. Since ToxR protein abundance is correlated with increased *ompU* expression in nutrient limiting conditions [32], we assessed if the loss of RND-mediated efflux affected ToxR protein abundance. ToxR Western blots revealed similar levels of ToxR production in WT and JB485 (Fig 2C) and their isogenic ΔPPD mutants (Fig 3C). Similarly, deletion of the PPD in WT did not affect *aphA*, *toxT*, *ctxA*, and *tcpA* expression (S5 Fig), as previously reported [31]. Together these results confirmed that the *toxR*^ΔPPD^ allele was functional in WT and JB485 and indicated that the PPD was dispensable for *ompU* expression and ToxR regulon expression.

We next quantified *leuO* expression in the above strains. The results showed increased *leuO* transcription in JB485 relative to WT and impaired *leuO* expression in the isogenic Δ*toxRS* mutants of both strains (Fig 3E); confirming that *toxR* positively regulated *leuO* expression in JB485. In contrast, deletion of the ToxR PPD in WT resulted in a ~5-fold reduction in *leuO* expression, indicating that the PPD differentially affects ToxR activity at the *ompU* and *leuO* promoters. Deletion of the PPD in JB485 did not abolish *leuO* expression, but rather reduced *leuO* expression to WT levels (Fig 3E). This suggests that ToxR lacking its PPD (i.e. ToxR^ΔPPD^) maintains the capacity to activate *leuO* expression in JB485, but not in WT. From these results, we concluded that the ToxR PPD is important for *leuO* transcription including increased *leuO* expression in cells lacking RND efflux. However, since *leuO* was still expressed in JB485*toxR*^ΔPPD^, albeit at a reduced level, other factors also influence *leuO* expression in the absence of RND efflux.

### Malate induces *leuO* transcription via ToxR

**Fig 4:**
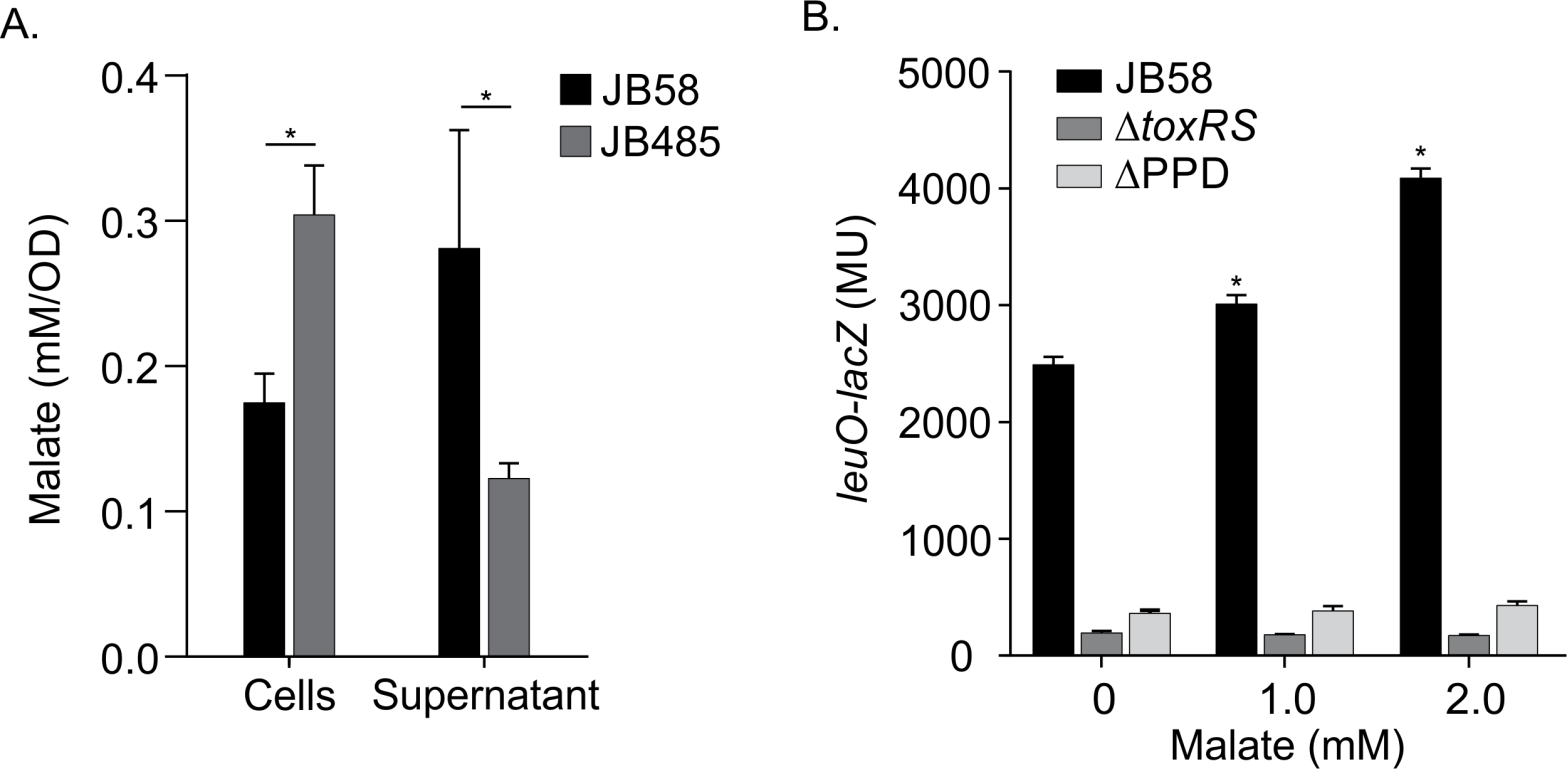
ToxR activates *leuO* transcription in response to malate. (A) Malate quantification in WT and RND null mutant JB485 culture supernatants and cells following growth under AKI conditions for 3.5h. Malate was quantified as described in the methods. The reported results are the means ± SD of six biological replicates with each replicate consisting of two technical replicates. * P≤0.05. (B) The indicated *V*. *cholerae* strains bearing pXB266 (*leuO-lacZ*) were cultured under AKI conditions for 4h when malate was added to 0, 1 or 2 mM. The cultures were then incubated with shaking for an additional hour when β-galactosidase production was quantified. The data are the mean ±SD of 3 independent experiments. * P≤0.05 relative to the no malate control.

The above data suggested that ToxR was activated upon loss of RND efflux. From this we speculated that cell metabolites accumulated in in the RND negative strain JB485 and were responsible for the changes in ToxR activity and increased *leuO* expression. The RNAseq results revealed differential expression of genes involved in malate metabolism with *fumC* (fumarate hydratase) being downregulated (S1 Table) and *mdh* (malate dehydrogenase) being upregulated by ~1.6-fold. As gene expression can be regulated by product feedback, this suggested that malate may accumulate in JB485 to effect ToxR activation. To test this, we quantified malate in JB58 and JB485 cells and culture supernatants following growth under AKI conditions. The results showed differential malate accumulation between WT and JB485. Cell lysates of strain JB485 contained elevated levels of malate relative to WT whereas WT culture supernatants contained more malate than JB485 supernatants (Fig 4A). These results supported the hypothesis that the absence of RND-mediated efflux resulted in intracellular malate accumulation which could be influencing ToxR activity in JB485.

We next asked if the addition of exogenous malate stimulated ToxR-dependent expression of *leuO*. To test this, we quantified *leuO-lacZ* expression in WT, *toxR*^ΔPPD^ and Δ*toxRS* following growth under AKI conditions for 1hr in the presence of varying concentrations of malate. We found that malate addition did not affect *leuO* expression in the *toxR*^ΔPPD^ and Δ*toxRS* mutants, but increased *leuO* expression in a concentration-dependent manner in WT (Fig 4B), suggesting that malate can influence the activation state of ToxR by a process that was dependent upon the ToxR PPD. The effects of exogenous malate on *leuO* expression in WT were somewhat reduced relative to what was observed in JB485. The reasons for this are unclear, but could reflect physiological differences in WT that limit periplasmic malate accumulation in WT including active efflux by the RND systems and the production of the cation-selective porin OmpU which could restrict malate diffusion across the outer membrane [33]. When this assay was repeated with fumarate we did not observe changes in *leuO* expression (S6 Fig), suggesting that the effects of malate on ToxR were not the due to nonspecific effects of C4 dicarboxylic acids on the cell.

### RND efflux is essential for colonization

The RND null mutant JB485 was highly attenuated in the infant mouse model and was not recovered from the small intestine following challenge [14]. Since the loss of RND-mediated efflux was pleiotropic, with cells exhibiting antimicrobial hypersensitivity and attenuated virulence factor production, the relative contribution of these two phenotypes to colonization was unclear. As the studies here revealed that *leuO* deletion restored in vitro virulence factor production in JB485, we tested whether *leuO* deletion also restored the ability of JB485 to colonize infant mice. To test this, we performed colonization competition assays between JB485Δ*leuO* and WT. The results showed that neither JB485Δ*leuO* nor JB485 could be recovered from the intestine of challenged mice, further confirming that the RND transporters were essential for colonization. We considered the possibility that deletion of *leuO* may have dysregulated virulence factor production in vivo and thus led to a colonization defect. To address this, we performed a competition assay between JB485 and JB485*toxR*^ΔPPD^; ToxR^ΔPPD^ mutants have been shown to activate virulence factors normally (S5 Fig and [31]). However, we were again unable to recover JB485 or JB485*toxR*^ΔPPD^ from the challenged mice. Taken together, these results suggest that the function of the RND transporters in antimicrobial resistance, and/or their contribution to unknown physiological functions, is likely epistatic to virulence factor production for colonization.

## Discussion

The broad effects of RND efflux on the *V*. *cholerae* transcriptome suggested that RND-mediated efflux impacts multiple aspects of bacterial physiology. It was noteworthy that 5 of the 6 RND operons (i.e. *vexRAB*, *vexCD*, *vexGH*, *vexIJK*, and *vexLM*), the two local RND regulators *breR* and *vexR*, and *tolC*, were upregulated in JB485. This suggests that the RND efflux systems autoregulate their own expression. While the mechanism for this remains unclear, RND systems are usually regulated in response to their substrates. From this we infer that cell metabolites are accumulating in the absence of RND efflux and activating RND systems expression via a feedback mechanism. This conclusion is bolstered by reports showing that biosynthetic pathway mutants, which are predicted to accumulate metabolic intermediates, induce RND transporter expression in *V*. *cholerae* and *E*. *coli* [23, 34]. These findings also support the conclusion that a native function of at least some of the RND transporters is to maintain cell homeostasis by removing potentially toxic metabolites from within the cell.

One of the most highly upregulated genes in the RND-negative mutant was *leuO*. *leuO* expression is regulated by ToxR in response to environmental cues [11, 12, 25–27]. Genetic studies suggest that *leuO* expression is induced by small molecules interacting with the ToxR PPD [11, 12]. Here we showed that ToxR-mediated *leuO* expression in the absence of RND efflux was dependent upon the ToxR PPD. We documented that *leuO* expression increased in the absence of RND-mediated efflux, while the amount of ToxR protein remained the same. This indicated that ToxR was activated in the absence of RND-mediated efflux, and that this process was dependent upon the ToxR PPD. These findings alluded to the possibility that cell metabolites may influence ToxR activity. The fact that malate addition enhanced *leuO* expression via a ToxR PPD-dependent process supported this hypothesis. Based on these data we propose that ToxR can function as a metabolic sensor. Previous reports, together with this study, have shown that ToxR can respond to multiple metabolites (e.g. cyclic dipeptides, bile salts and malate) via its PPD, suggesting that ToxR may be promiscuous in agonist recognition. Exactly how ToxR can sense multiple agonists is unknown. However, recent studies showing that destabilization of the PPD promoted ToxR activation provides an intriguing model that could accommodate multiple agonists [10].

In this study, we documented that exogenous malate stimulated *leuO* expression by a process that was dependent on the ToxR PPD. We also documented increased cell-associated malate in RND deficient *V*. *cholerae*. Taken together these results suggest that malate might function as a ToxR agonist in RND deficient cells and that malate may be a substrate for the RND transporters. It is interesting to note that malate was shown to repress *toxT* expression in classical biotypes while TCA cycle mutants that diminished malate production (i.e. fumarase mutants) exhibited increased *toxT* expression [35]. While the mechanism responsible for these phenotypes is unclear, it is tempting to speculate that the effects of malate on *toxT* expression were mediated through malate-dependent effects on ToxR activation as described here. Malate is produced in the cytoplasm and thus needs to be transported to the periplasm to access the ToxR PPD. The mechanism by which this might occur is unknown. Malate efflux systems belonging to PET (Putative Efflux Transporter) family are present in plants and homologs of these efflux systems are found in many bacteria [36, 37], but we were not able to identify PET homologs in *V*. *cholerae*. It is unclear if malate was the sole agonist that was responsible for increased *leuO* expression in RND-deficient cells. The fact that the magnitude of *leuO* induction in WT cells treated with malate was lower than what was observed in JB485 could suggest that other metabolites also contributed to ToxR activation in RND deficient cells. For example, it is possible that malate indirectly affects ToxR activation by a feedback mechanism. If malate was an efflux substrate, it could serve as a competitive efflux inhibitor and thus alter the export of a second metabolite. Malate accumulation could also alter cell metabolism and lead to the generation of other unknown metabolites that serve as ToxR agonists, or that malate could function cooperatively with other metabolites (which may be limiting in WT) to affect the activation state of ToxR. Discrimination between these possibilities will require further work.

**Fig 5:**
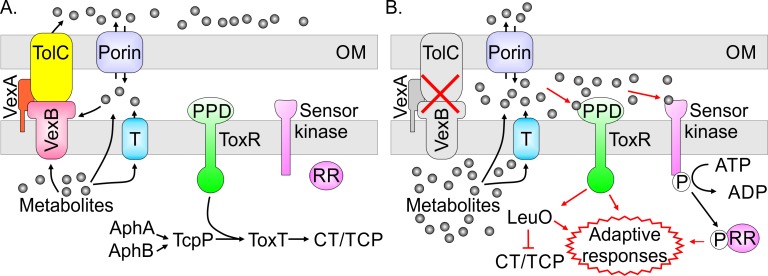
Putative model for RND-mediated efflux in environmental sensing. (A) In WT cells growing under virulence gene inducing conditions the RND transporters expel metabolites from within the periplasm and ToxR functions with TcpP to activate ToxR regulon expression. Metabolites which are produced in the cytoplasm enter the periplasmic compartment either by passive diffusion through the cytoplasmic membrane or by active transport systems localized in the cytoplasmic membrane (denoted by the blue box). Exogenous metabolites enter the periplasm by diffusion through porin channels. (B) Inhibition of RND-mediated efflux results in the accumulation of metabolites that are normally expelled by the RND transporters. The accumulated metabolites activate ToxR via interaction with its periplasmic domain. Activated ToxR then modulates target gene expression (e.g. *leuO*) which leads to virulence repression and altered cell physiology. The accumulated metabolites also activate select two-component regulatory systems (e.g. CpxRA, CarRS, OmpREnvZ and VieSAB) which further effect transcriptional responses and environmental adaptation. Abbreviations: CT, cholera toxin; OM, outer membrane; PPD, periplasmic domain, RR, response regulator, TCP, toxin coregulated pilus; T, metabolite transporter.

The link between RND efflux and ToxR activity suggests a novel paradigm for RND-mediated efflux in sensing and responding to environmental cues (Fig 5). Under normal conditions, the RND transporters maintain homeostasis by exporting metabolites from the cytoplasm and periplasm (Fig 5A). In cells with impaired RND efflux, metabolites accumulate within the cell and interact with environmental sensors like ToxR to stimulate the expression of adaptive responses (Fig 5B). In the case of ToxR, this results in elevated *leuO* expression and the subsequent repression of CT and TCP production. It is unclear how metabolites affect ToxR activation, but a recent report showing that bile salts enhance ToxR activity by binding to the PPD to promote heterodimer formation with ToxS provides a compelling model by which this could occur [10]. While increased *leuO* transcription serves as a marker for ToxR activation, the loss of RND efflux clearly affected several other environmental sensing systems. This is evidenced by the fact that 17 out of the 30 differentially expressed regulatory genes belong to TCSs. This included the CpxRA system where 14 of the 25 genes in the Cpx regulon were differentially expressed in the RND mutant [18]; a finding consistent with a recent study showing that vibriobactin secretion by VexGH RND system was required to maintain the CpxRA system in an inactive state [4]. The preferential activation of TCSs in cells lacking RND efflux supports the conclusion that the RND systems influence homeostasis by effluxing cell metabolites.

The above model suggests the intriguing possibility that RND efflux systems may affect in vivo gene expression in response to host-derived compounds. For example, upon ingestion *V*. *cholerae* encounters high concentrations of bile salts in the lumen of the small intestine. Since bile salts are major RND transporter substrates, they could serve as competitive inhibitors for metabolite export and thus affect metabolite accumulation within the cell. Metabolites likely also accumulate late in infection, when *V*. *cholerae* is present at high cell titers in the small intestine, and may affect transcriptional responses via periplasmic sensors. A number of late infection phenotypes are important for *V*. *cholerae* pathogenesis including repression of the ToxR regulon and the expression of dissemination and transmission phenotypes (reviewed in [38]). While the regulation of these phenotypes remains unknown, the expression of *leuO* late during infection [12], and the suppression of virulence factor production in human and animal shed *V*. *cholerae* [39, 40], argue that the proposed model may be relevant in vivo. This model could also be extended to other Gram-negative bacteria where the RND transporters have broad effects on physiology and virulence [2, 3].

RND efflux deficient *V*. *cholerae* exhibited decreased *tcpPH* expression, reduced CT and TCP production, and a colonization defect in the infant mouse model [12]. Here we show that the defect in CT/TCP production was due to increased *leuO* expression as evidenced by the observation that deletion of *leuO* in JB485 restored *tcpPH* expression and CT and TCP production. This provided additional evidence to support the novel conclusion that ToxR can function as a virulence repressor. Interestingly, *leuO* deletion did not restore the ability of RND mutant strain JB485 to colonize infant mice. The reasons for this are unclear, but may be related to increased sensitivity of JB485 to antimicrobial compounds that are abundant in the intestine including CAPs, bile salts and other detergent-like molecules. It is also possible that the loss of RND efflux imparted unknown effects on cell physiology that negatively impacted *V*. *cholerae* pathogenesis or resulted in dysregulation of virulence gene expression in vivo. For example, dysregulated TCP production would impact the ability of *V*. *cholerae* to access colonization niches in the intestine or to adhere to the intestinal epithelium. Overall our results support the conclusion that RND efflux has pleiotropic effects on pathogenesis; being required for virulence gene expression, resistance to antimicrobial compounds that are present in the host, and perhaps other unknown aspects of pathogenesis.

## Materials and methods

### Bacterial strains and growth conditions

The bacterial strains used in this study are listed in Table 2. *E*. *coli* strain EC100λpir was used for cloning experiments. *E*. *coli* strain SM10λpir was used for conjugating plasmids into *V*. *cholerae*. Bacterial strains were routinely grown in Lysogeny Broth (LB) broth or on LB agar at 37°C. AKI growth conditions were used to induce virulence gene expression in *V*. *cholerae* as previously described [41]. Antibiotics were used at the following concentrations: carbenicillin (Cb), 100 μg/ml; kanamycin (Km), 50 μg/ml; and streptomycin (Sm), 100 μg/ml.

**Table 2:** Strains and plasmids used in this study.

Strains		Characteristics	Source
***E*. *coli***			
	EC100D pir+	*F- mcrA Δ(mrr-hsdRMS-mcrBC) φ80dlacZΔM15 ΔlacX74 recA1 endA1 araD139 Δ(ara*, *leu)7697 galU galK λ- rpsL (StrR) nupG pir+(DHFR)*	Epicenter
	SM10λpir	*thi-1 thr leu tonA lacY supE recA*::*RP4-2-Tc*::*Mu Km*^*r*^ *(λ pirR6K)*	[45]
***V*. *cholerae***			
	JB3	*V*. *cholerae* O1 El Tor strain N16961, Sm^r^	Lab collection
	JB58	*V*. *cholerae* O1 El Tor N16961, Sm^r^ *ΔlacZ*	Lab collection
	JB485	JB58 Δ*vexB* Δ*vexD* Δ*vexF* Δ*vexH* Δ*vexK* Δ*vexM*	[14]
	DK296	JB485 *lacZ*^+^	This study
	XBV222	JB58 Δ*leuO*	[12]
	XBV255	JB485 Δ*leuO*	This study
	JB495	JB58 Δ*vexB*	[14]
	JB692	JB58 Δ*vexD*	[14]
	JB694	JB58 Δ*vexB* Δ*vexD*	[14]
	JB464	JB58 Δ*vexD* Δ*vexF* Δ*vexH* Δ*vexK* Δ*vexM*	[14]
	JB740	JB58 Δ*vexB* Δ*vexD* Δ*vexK*	[14]
	DT12	JB58 Δ*vexB* Δ*vexD* Δ*vexH* Δ*vexK*	[28]
	DT30	JB58 Δ*vexB* Δ*vexD* Δ*vexH*	[28]
	DT422	JB58 Δ*vexB* Δ*vexF* Δ*vexH* Δ*vexK* Δ*vexM*	[28]
	DT733	JB58 Δ*toxRS*	[23]
	XBV148	JB58 Δ*aphB*	[25]
	XBV332	JB485 Δ*toxRS*	This study
	SS4	JB58 *toxR*^ΔPPD^	[11]
	XBV468	JB485 *toxR*^ΔPPD^	This study
**Plasmids**			
	pBAD18Km	Arabinose regulated expression plasmid, Km^R^	[46]
	pBAD24	Arabinose regulated expression plasmid, Cb^R^	[46]
	pBAD33	Arabinose regulated expression plasmid, Cm^R^	[46]
	pBAD-*tcpPH*	pBAD24 expressing *tcpPH*	[47]
	pBAD-*toxT*	pBAD24 expressing *toxT*	[47]
	pCM10	Lux-based transcriptional reporter plasmid, Cm^R^	[48]
	pCM10-*leuO*	pCM10 containing the *leuO* promoter	[12]
	pTL61T	*lacZ* transcriptional reporter plasmid, Cb^R^	[49]
	pXB192	pTL61T containing the *toxT* promoter	[14]
	pXB193	pTL61T containing the *ctxAB* promoter	This study
	pXB194	pTL61T containing the *tcpA* promoter	This study
	pXB202	pTL61T containing the *aphA* promoter	[50]
	pAL144	pTL61T containing the *ompU* promoter	[50]
	pWM91-*lacZ*	Allelic exchange vector for repairing the *lacZ* locus in *Vibrio cholerae*	This study
	pWM91-Δ*leuO*	Allelic exchange vector for in-frame deletion of *leuO*	[27]
	pWM91-Δ*toxRS*	Allelic exchange vector for in-frame deletion of *toxRS*	[39]
	pWM91-*toxR*^ΔPPD^	Allelic exchange vector for in-frame deletion of the ToxR PPD	[11]
	pXB208	pBAD18Km expressing *aphA*	[51]
	pXB209	pBAD18Km expressing *aphB*	This study
	pXB302-2	pBAD33 containing the *toxRS* genes	[11]
	pXB286	pBAD18 containing the *toxR*^ΔPPD^*S* genes	[12]
	pXB266	pTL61T containing the *leuO* promoter	[12]
	pXB269	pBAD18Km expressing *leuO*	[12]
	pVA126	pBAD33 expressing *leuO*	[25]
	pVA195	pTL61T containing the *rtxB* promoter	This study

### Plasmid and mutant construction

The plasmids and oligonucleotides used in this study are listed in Table 2 and S3, respectively. Chromosomal DNA from strain N16961 was used as the template for cloning experiments. Transcriptional reporter plasmids were constructed as described below. The promoter regions of *ctxAB*, *tcpA* and *rtxB* were generated by PCR using the *PctxAB*-F/*PctxAB*-R, *PtcpA*-F/*PtcpA*-R, or *PrtxB*-F/*PrtxB*-R oligonucleotide primers. The resulting amplicons were digested with XhoI and XbaI restriction endonucleases and ligated into similarly digested pTL61T to generate the plasmids pXB193, pXB194 and pVA195, respectively. pXB209 was constructed similarly using the *aphB*-F/*aphB*-R primers with the resulting PCR amplicon being digested with EcoRI and XbaI restriction endonucleases before being ligated into similarly digested pBAD18Km. Deletion of *leuO* and the *toxR* PPD domain was generated using the plasmid pWM91Δ*leuO* or pWM91::Δ*toxRppd* as previously described [11, 12]. Repair of *lacZ* in JB485 was accomplished by using PCR to amplify the *lacZ* locus from N16961 strain JB3 using the Vc.*lacZ*.F.BamHI and Vc.*lacZ*.R.SacI PCR primers. The resulting 5.1 kb amplicon, containing the *lacZ* locus and ~1 kb of flanking DNA sequence, was digested with BamHI and SacI restriction endonucleases before being ligated into similarly digested pWM91 to generate pWM91-*lacZ*. The resulting plasmid was then used to repair the *lacZ* allele by allelic exchange as previously described [11, 12].

### Quantitative real time PCR

*V*. *cholerae* strains were grown under AKI conditions in AKI broth. Total RNA was then isolated from the cultures at 3h using Trizol per the manufacturer’s directions. cDNA was generated from the total RNA using the Superscript III RT (Invitrogen) and used as a template for qRT-PCR with the SYBR Green Dye Kit (Fisher Scientific). The expression level of specific genes was quantified by amplifying 25 ng cDNA with 0.3 μM primers using the SYBR green PCR mix on a StepOnePlus real-time PCR System (Applied Biosystems). The gene encoding for DNA gyrase (*gryA*) was used as the internal control. Gene expression changes were calculated using the 2^−ΔΔCT^ method and the presented results are the means ± standard error of the means (SEM) from three biological replicates, with each biological replicate generated from three technical replicates.

### RNA sequencing

Total RNA from *V*. *cholerae* strains JB58 and JB485 grown under AKI conditions for 3h were isolated using TRIzol per the manufacturer’s directions (Invitrogen) and further purified using an RNeasy kit with in column DNase treatment (Qiagen). The resulting RNA samples were assessed using a Qubit 2.0 fluorimeter (Thermo Scientific) and Agilent Tapestation 2200 (Agilent Technologies). Sequencing libraries were generated using the Illumina TruSeq RNA Access library prep kit (Illumina). Cluster generation and 75-bp single-read single-indexed sequencing was performed on Illumina NextSeq 500 (Illumina). The resulting raw reads were trimmed to remove adaptor/primer sequences. CLC Genomics Workbench version 10.1 (Qiagen) was then used to map the reads from three independent experiments to the N16961 genome [42]. The identification of differentially expressed genes was accomplished using the CLC Genomics Workbench RNA-Seq Analysis tool. Genes showing a 2-fold or greater difference in expression and a P-value and False Discovery Rate P-value of less than or equal to 0.05 were identified as differentially expressed genes. The RNA sequencing data was deposited in the National Center for Biotechnology Information Sequence Read Archive under accession number SRP109296.

### Transcriptional reporter assays

Expression of promoters that were transcriptionally fused to *lacZ* were assayed following growth under AKI conditions. Samples from the cultures were taken at the indicated time points and processed in triplicate to assay for β-galactosidase activity as previously described [43]. The reporter experiments were independently performed at least three times. Statistical analyses were determined using the Student’s t-test. The concentrations of malate and fumarate used in the reporter assays was based on the physiological concentrations reported for *E*. *coli* (i.e. 1.7 mM for malate and 0.12 mM for fumarate) [44].

### Characterization of CT, TcpA, AphB, ToxR and TcpP production

CT production in *V*. *cholerae* strains grown under AKI conditions was determined by GM1 enzyme-linked immunosorbent CT assays as previously described using purified CT (Sigma) as a standard [28]. The production of TcpA, ToxR, AphB and TcpP was determined respectively by Western immunoblotting as previously described using the polyclonal rabbit antisera against TcpA, ToxR, AphB, and TcpP [12]. Immunoreactive proteins on the Western blots were visualized using the SuperSignal West Pico chemiluminescent detection kit (Thermo Scientific).

### Electrophoretic mobility shift assay (EMSA)

LeuO was purified from middle logarithmic phase LB broth cultures of *E*. *coli* ER2566 bearing pVA175 (pMAL-c2::*leuO)* following induction with isopropyl β-D-1-thiogalactopyranoside (0.3 mM) for 2h as previously described [11, 25]. The cells were then harvested by centrifugation, resuspended in column buffer (20 mM Tris-HCl, 200 mM NaCl, 1 mM EDTA, 1 mM phenylmethylsulfonyl fluoride), and lysed using a M-11P Microfluidizer according to the manufacturer’s instructions (Microfluidics). The lysates were cleared by centrifugation at 15,000 x g for 20 min and the clarified lysates were diluted 1:6 with column buffer and run over an amylose resin chromatography column (New England Biolabs). Proteins bound to the amylose resin were then eluted with elution buffer (20 mM Tris-HCl, 200 mM NaCl, 1 mM EDTA, 10 mM maltose), before LeuO was liberated from the MBP using factor Xa protease. Protein purity was assessed by SDS–PAGE with Coomassie brilliant blue R-250 staining. Protein concentration was determined using the Bradford Assay kit according to the manufacturer’s instructions (Thermo Scientific).

The gel-shift assays were performed as previously described [25]. Briefly, biotinylated probes were generated by PCR using primers that were purchased from the manufacturer (IDT) with a 5’ biotin label. The biotinylated probes were gel purified before being used in the gel shift assays. The DNA binding reactions were performed in a final volume 10 μl of binding buffer (10 mM Tris-HCl (pH 7.4), 5 mM NaCl, 50 mM KCl, 1 mM EDTA, 50 μg/ml BSA, 1.5 nM biotinylated probe and 10 μg/ml sheared salmon sperm DNA). The binding reactions were incubated at 30°C for 30 minutes before being subjected to electrophoresis on non-denaturing 5% polyacrylamide TBE gels. The resolved gels were electroblotted to positively charged nylon membrane before the membranes were subjected to UV crosslinking at 120,000 microjoules using a Stratalinker 1800 Crosslinker (Strategene). The biotinylated probes were then detected using the Pierce Chemiluminescent Nucleic Acid Detection Module (Thermo Scientific) and visualized using a FluorChem E imaging system (Protein Simple).

### Malate determination

JB58 and JB485 were cultured under AKI conditions for 3.5h when the culture optical density at 600 nm was recorded and a one mL culture aliquot was collected. The cells were separated from the supernatant by centrifugation and the supernatant and cell pellet retained. Cell lysates were generated by resuspending the cell pellet in one mL of ddH_2_O before subjecting the cells to three freeze-thaw cycles followed by sonication. Aliquots of the culture supernatant and cell lysates were then assayed for malate using the Malate Assay Kit (Sigma) according to the manufacturer’s directions and normalized by optical density. The reported results are the means ± standard deviation from six biological replicates, with each biological replicate generated from two technical replicates.

### Infant mouse competition assay

Cultures of XBV255 (JB485Δ*leuO*Δ*lacZ*) and WT strain JB3 (N16961 *lacZ* positive) or DK296 (RND null, *lacZ* positive) and XBV468 (JB485 ΔPPD Δ*lacZ*) were combined at a 1:1 ratio before being delivered perorally to infant mice as previously described [14]. The following day the small intestine from the stomach to the cecum was excised and homogenized in phosphate buffered saline. Serial dilutions of the homogenates were then plated onto LB agar containing X-gal and streptomycin to enumerate the bacterial strains based on colony color. A competitive index was then calculated as the ratio of the test strains in the input divided by the ratio of test strains in the output.

### Ethics statement

Animal protocols were approved by the University of Pittsburgh Institutional Animal Care and Use Committee (Protocol number 15015310) and met the standards for humane animal care and use as set by the Animal Welfare Act and the NIH Guide for the Care and Use of Laboratory Animals.

## Supporting information

S1 Fig Loss of RND-mediated efflux activates *leuO* expression. The indicated *V*. *cholerae* RND efflux mutants bearing *leuO-lacZ* reporter plasmid pXB266 were cultured under AKI conditions for 5h when β-galactosidase activity was quantified. The data are means ± SD from at least three independent experiments. Statistical significance was determined using one-way ANOVA, comparing the means to WT. *, P<0.05.
(TIF)

S2 Fig Complementation of CT production in JB485. (A & B) WT and JB485 containing pBAD24 or pBAD18Km expressing, *toxT*, *tcpPH*, *aphA*, *aphB* or *leuO* were grown under AKI conditions in the presence of the indicated concentration of arabinose overnight when CT production was quantified by a GM1 ELISA. P-values in panel A were determined using a Student’s t-test to compare the mean of the induced cultures with those of the no arabinose control; *, P<0.05. P-values for panel B were determined using a one-way ANOVA to compare the means to WT grown under the same condition; *, P<0.05.
(TIF)

S3 Fig AphB Western blot. The indicated strains were cultured under AKI conditions for 6h when culture aliquots were collected, normalized by optical density, and subjected to Western blotting using anti-AphB polyclonal antibody. AphB is indicated by the arrow. The nonspecific bands serve as loading controls.
(TIF)

S4 Fig LeuO binds to the *aphA* promoter. (A) Schematic of the *aphA* promoter showing the location of the probes used for the EMSA in panel B relative to the putative -35 and -10 elements. The schematic is not drawn to scale. (B) EMSA using purified LeuO and the biotinylated probes defined in panel A. The control probe is an arbitrarily selected internal fragment of the VCA1013 open reading frame.
(TIF)

S5 Fig The ToxR periplasmic domain is dispensable for expression of the ToxR regulon. WT, Δ*toxRS*, and *toxR*^ΔPPD^ strains carrying (A) *aphA-lacZ*, (B) *toxT-lacZ*, (C) *tcpA-lacZ*, or (D) *ctxAB-lacZ*, transcriptional reporter plasmids were cultured under AKI conditions for 5h when gene expression was quantified using a β-galactosidase assay. Data presented are the mean ±SD of three independent experiments. Statistical analysis was preformed using the Students t-test; *, P<0.05.
(TIF)

S6 Fig Fumarate does not affect *leuO* expression. *V*. *cholerae* strain JB58 bearing pCM10-*leuO* or the empty vector (pCM10) were cultured under AKI growth conditions for 4h when the indicated concentrations of fumarate were added. The cultures were then incubated with shaking for an additional hour before luciferase production was quantified using a Biotek Synergy 4 plate reader. Luciferase production is reported as relative light units (RLU) normalized by the optical density at 600 nm. The results are the mean ± SD of three independent experiments.
(TIF)

S1 Table Differentially expressed genes in RND deficient *V*. *cholerae*. (DOCX)

S2 Table Oligonucleotide primers used in this study. (DOCX)
