# Supplementary figures and images for: The Vibrio cholerae RND efflux systems impact virulence factor production and adaptive responses via periplasmic sensor proteins
Source: PLoS Pathog. 2018 Jan 5;14(1):e1006804. doi: 10.1371/journal.ppat.1006804 (PMC5773229; doi:10.1371/journal.ppat.1006804)

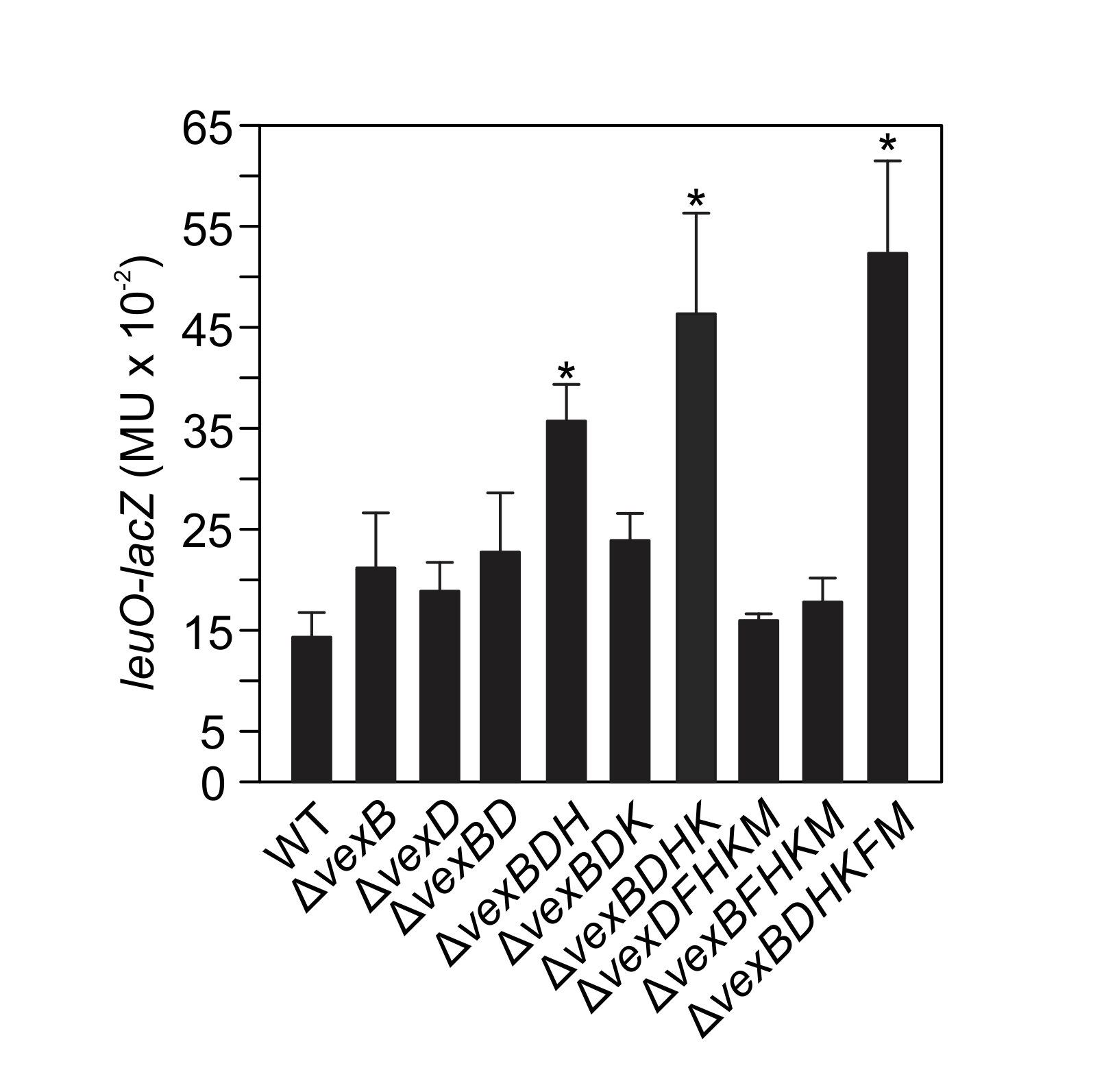

Supplement: S1 Fig — The indicated V. cholerae RND efflux mutants bearing leuO-lacZ reporter plasmid pXB266 were cultured under AKI conditions for 5h when β-galactosidase activity was quantified. The data are means ± SD from at least three independent experiments. Statistical significance was determined using one-way ANOVA, comparing the means to WT. *, P<0.05. (TIF) [file ppat.1006804.s001.tif]

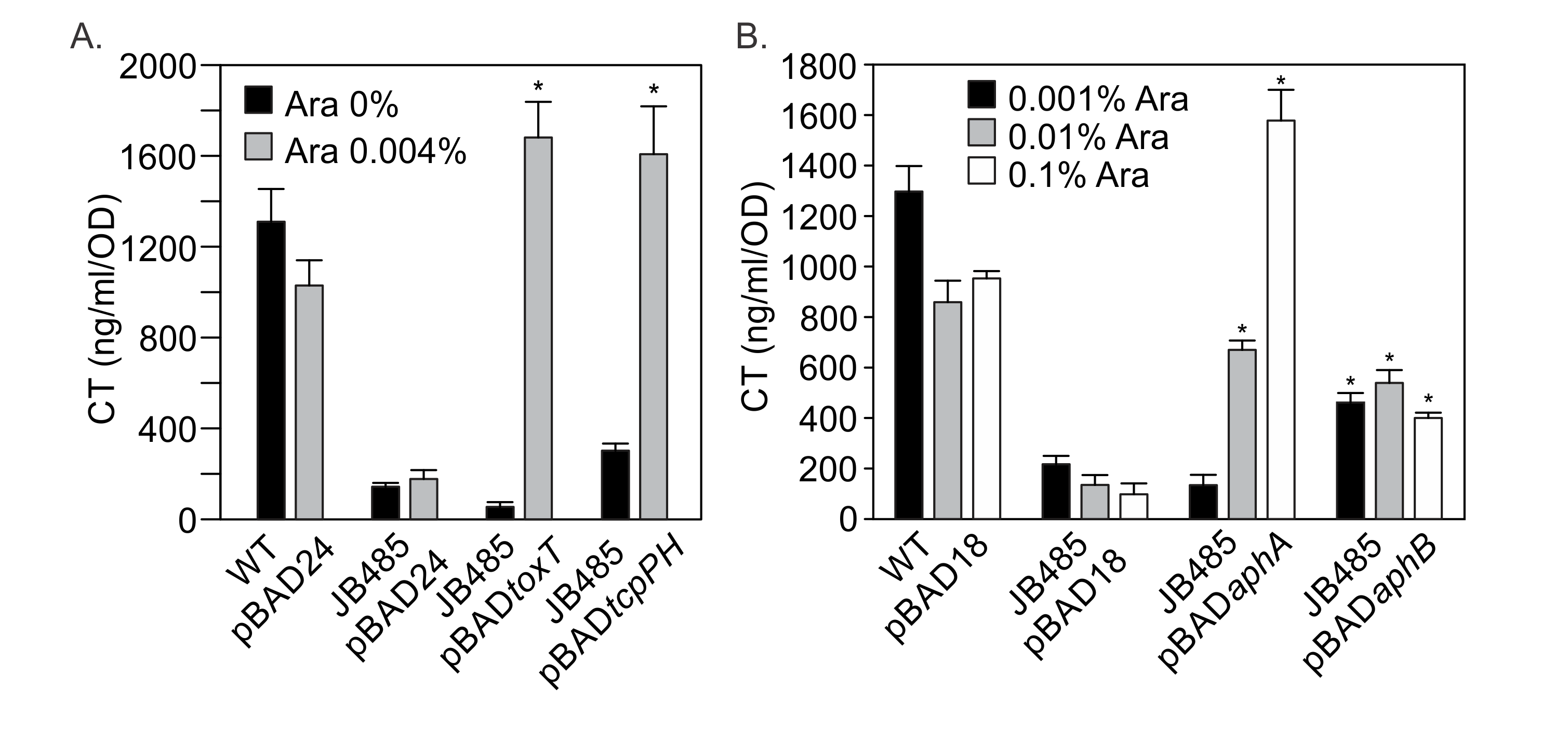

Supplement: S2 Fig — (A & B) WT and JB485 containing pBAD24 or pBAD18Km expressing, toxT, tcpPH, aphA, aphB or leuO were grown under AKI conditions in the presence of the indicated concentration of arabinose overnight when CT production was quantified by a GM1 ELISA. P-values in panel A were determined using a Student’s t-test to compare the mean of the induced cultures with those of the no arabinose control; *, P<0.05. P-values for panel B were determined using a one-way ANOVA to compare the means to WT grown under the same condition; *, P<0.05. (TIF) [file ppat.1006804.s002.tif]

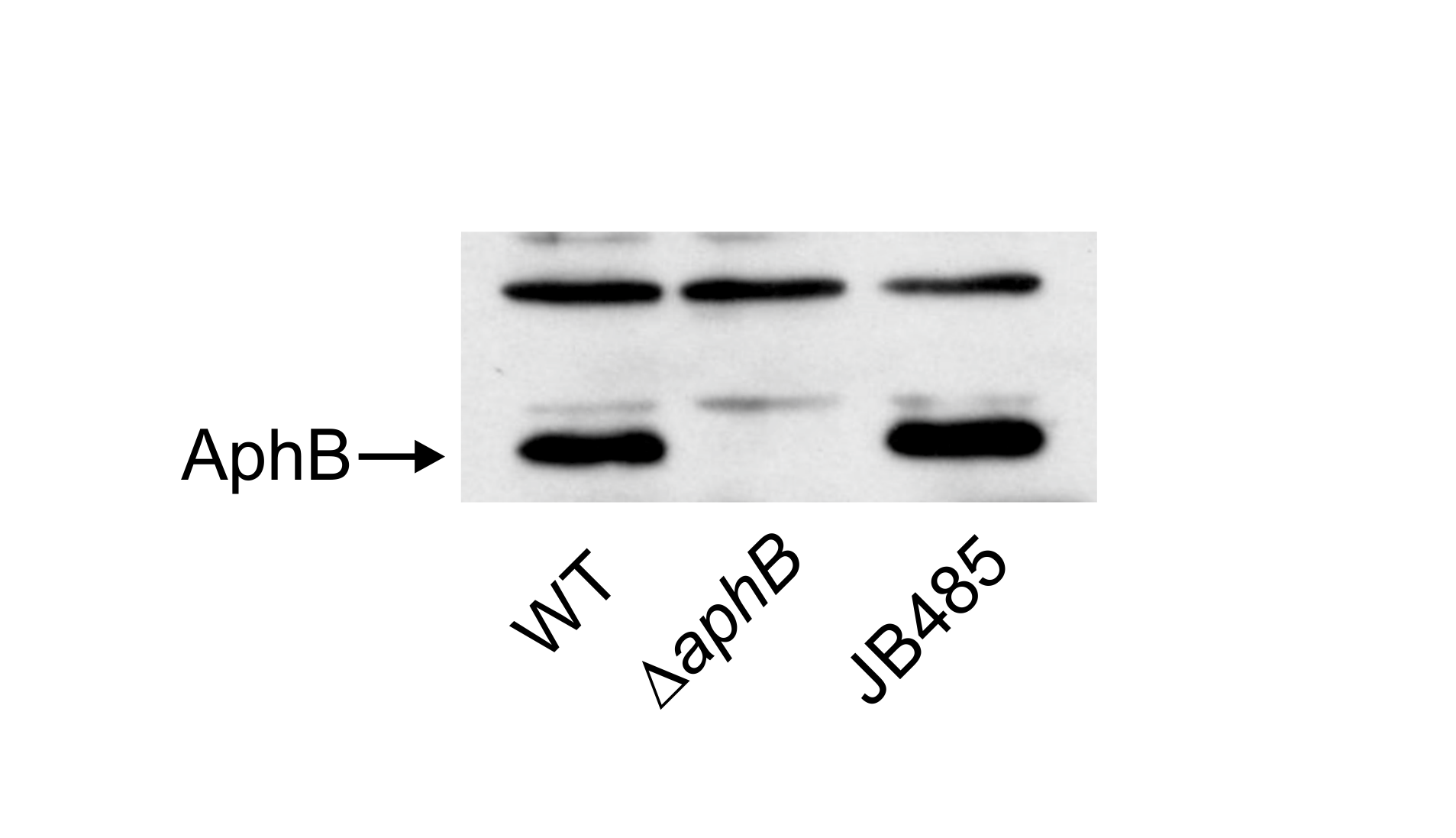

Supplement: S3 Fig — The indicated strains were cultured under AKI conditions for 6h when culture aliquots were collected, normalized by optical density, and subjected to Western blotting using anti-AphB polyclonal antibody. AphB is indicated by the arrow. The nonspecific bands serve as loading controls. (TIF) [file ppat.1006804.s003.tif]

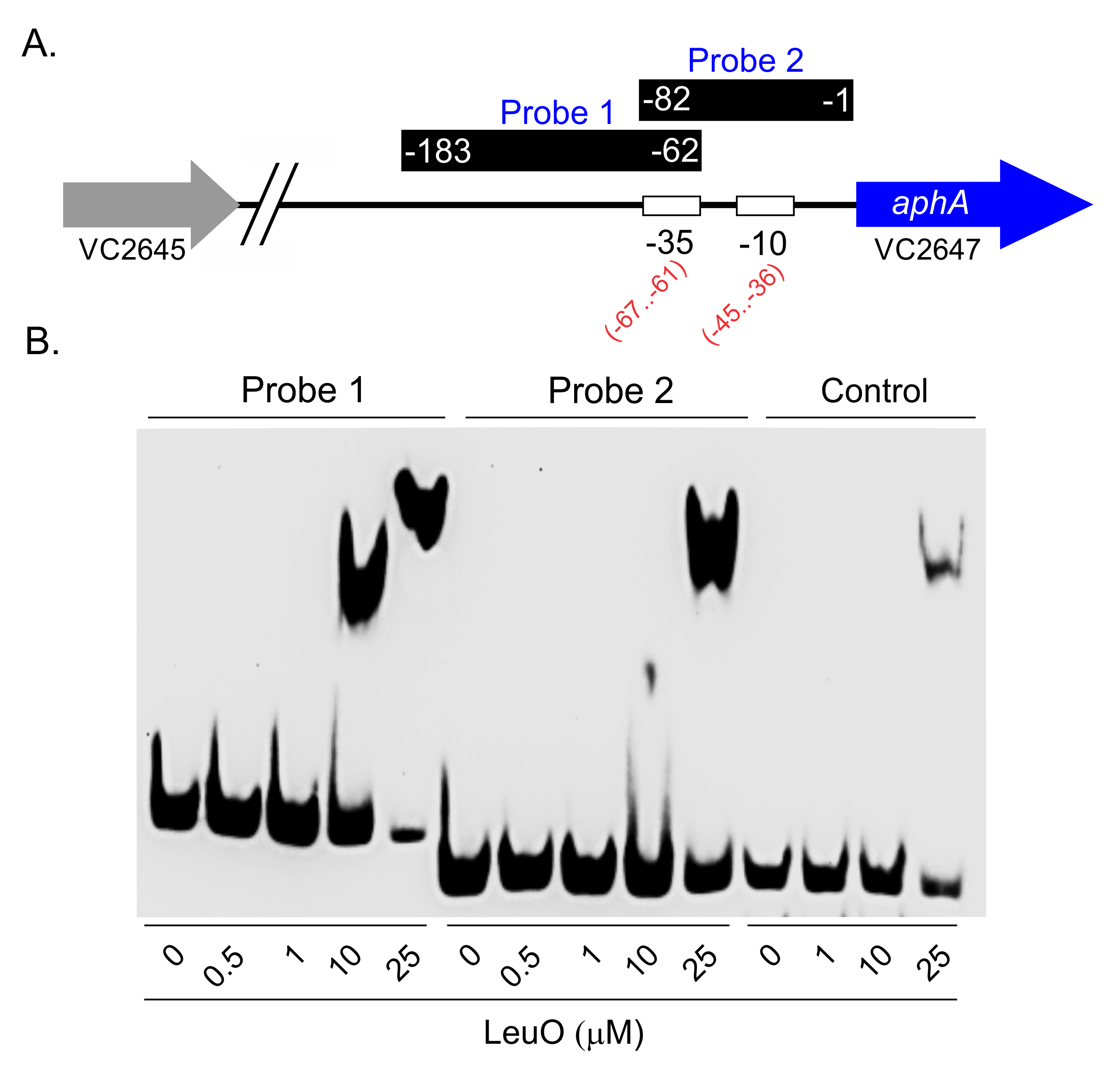

Supplement: S4 Fig — (A) Schematic of the aphA promoter showing the location of the probes used for the EMSA in panel B relative to the putative -35 and -10 elements. The schematic is not drawn to scale. (B) EMSA using purified LeuO and the biotinylated probes defined in panel A. The control probe is an arbitrarily selected internal fragment of the VCA1013 open reading frame. (TIF) [file ppat.1006804.s004.tif]

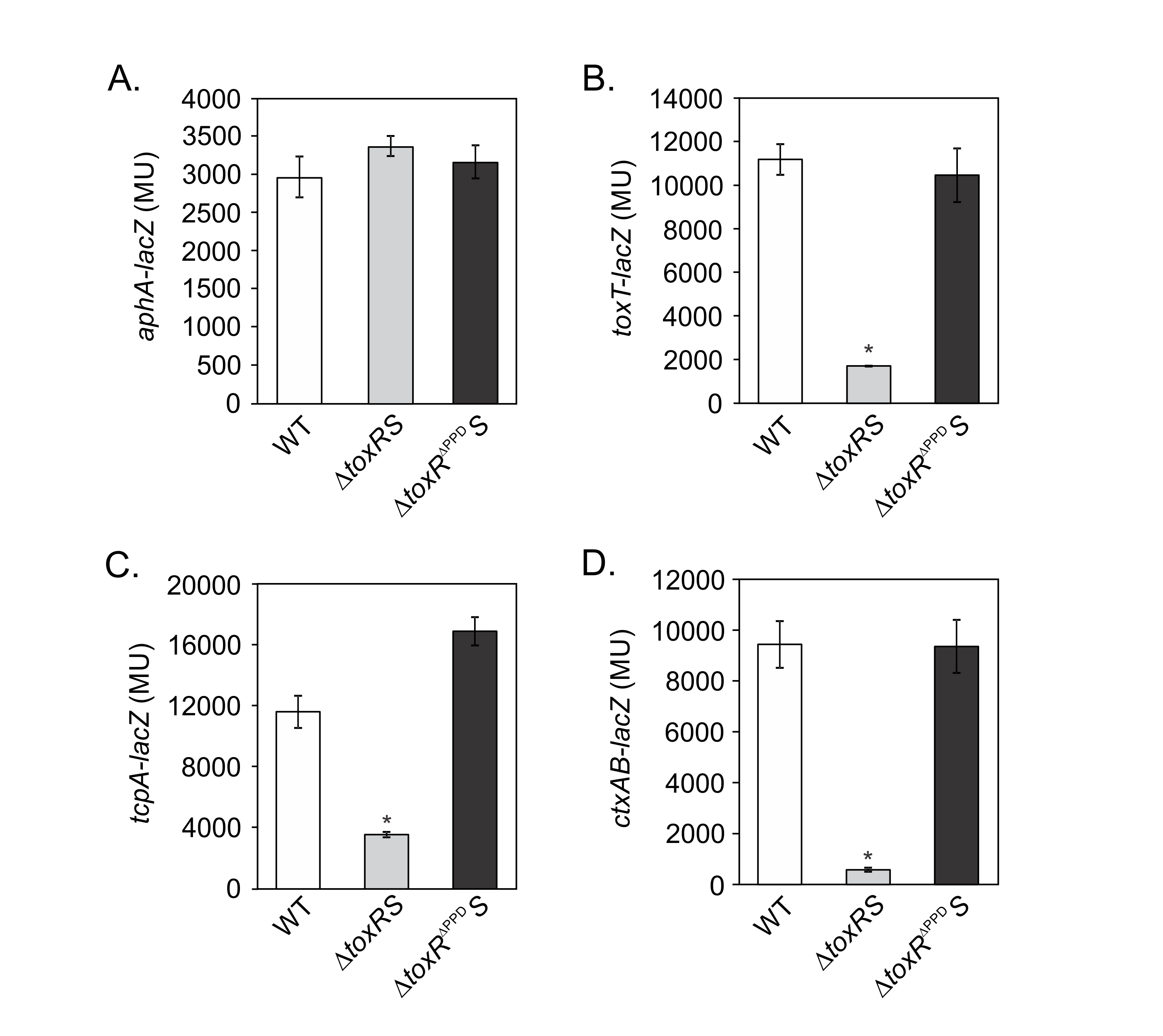

Supplement: S5 Fig — WT, ΔtoxRS, and toxRΔPPD strains carrying (A) aphA-lacZ, (B) toxT-lacZ, (C) tcpA-lacZ, or (D) ctxAB-lacZ, transcriptional reporter plasmids were cultured under AKI conditions for 5h when gene expression was quantified using a β-galactosidase assay. Data presented are the mean ±SD of three independent experiments. Statistical analysis was preformed using the Students t-test; *, P<0.05. (TIF) [file ppat.1006804.s005.tif]

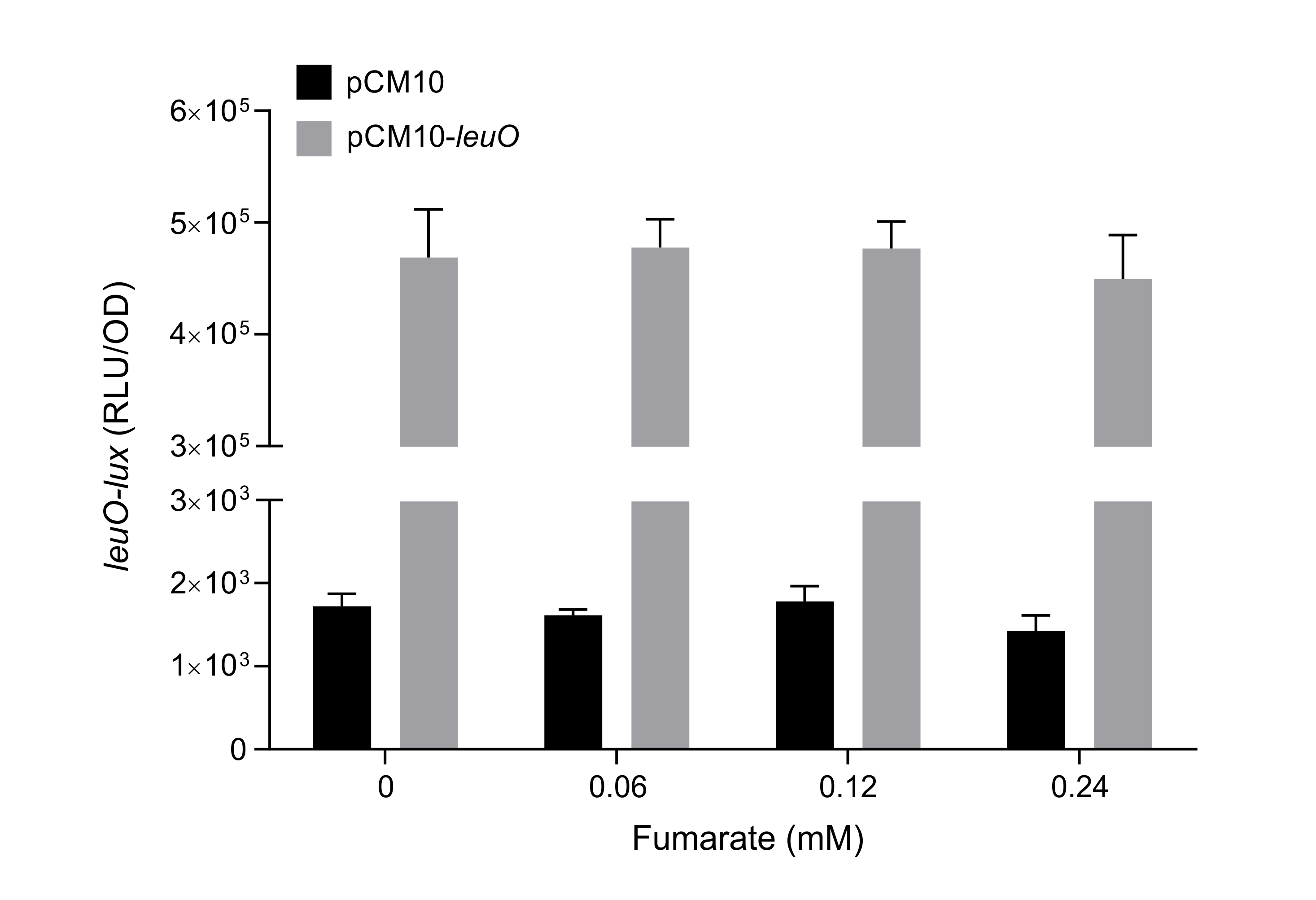

Supplement: S6 Fig — V. cholerae strain JB58 bearing pCM10-leuO or the empty vector (pCM10) were cultured under AKI growth conditions for 4h when the indicated concentrations of fumarate were added. The cultures were then incubated with shaking for an additional hour before luciferase production was quantified using a Biotek Synergy 4 plate reader. Luciferase production is reported as relative light units (RLU) normalized by the optical density at 600 nm. The results are the mean ± SD of three independent experiments. (TIF) [file ppat.1006804.s006.tif]
