# Supplementary material for: The Vibrio cholerae RND efflux systems impact virulence factor production and adaptive responses via periplasmic sensor proteins
Source: PLoS Pathog. 2018 Jan 5;14(1):e1006804. doi: 10.1371/journal.ppat.1006804 (PMC5773229; doi:10.1371/journal.ppat.1006804)
Supplement: S1 Table — (DOCX) [file ppat.1006804.s007.docx]

**Table S1. Differentially expressed genes in RND deficient *V. cholerae*.**

| **Locus number:** | **Gene ID:** | **Log2 fold change (JB485/WT):** | **P-value:** | **FDR p-value:** |
| --- | --- | --- | --- | --- |
| VC0008 | 2614084 | -1.133528691 | 3.57E-08 | 3.75E-07 |
| VC0009 | 2615705 | -1.103334022 | 7.54E-07 | 6.57E-06 |
| VC0010 | 2615295 | -0.994816533 | 9.62E-05 | 0.000584151 |
| VC0018 | 2614875 | 1.033868262 | 6.02E-05 | 0.000385987 |
| VC0027 | 2614461 | 1.308404517 | 4.16E-14 | 7.91E-13 |
| VC0028 | 2612960 | 1.672982429 | 0 | 0 |
| VC0029 | 2614456 | 1.501311303 | 0 | 0 |
| VC0030 | 2614457 | 1.540592994 | 6.11E-15 | 1.24E-13 |
| VC0031 | 2614464 | 1.723736524 | 0 | 0 |
| VC0076 | 2615765 | 1.238685474 | 0 | 0 |
| VC0110 | 2615141 | -1.321665784 | 0.002833267 | 0.011318943 |
| VC0133 | 2615826 | -1.564595986 | 5.14E-09 | 6.07E-08 |
| VC0165 | 2612954 | 3.618922061 | 0 | 0 |
| VC0166 | 2614082 | 4.032018164 | 0 | 0 |
| VC0167 | 2614083 | 1.036128433 | 1.95E-14 | 3.82E-13 |
| VC0184 | 2614437 | -1.130283073 | 5.97E-08 | 6.06E-07 |
| VC0188 | 2614711 | 1.277224619 | 8.30E-11 | 1.18E-09 |
| VC0199 | 2614283 | -1.806094209 | 0 | 0 |
| VC0200 | 2614270 | -3.217304774 | 0 | 0 |
| VC0201 | 2614271 | -1.686859694 | 1.40E-12 | 2.35E-11 |
| VC0202 | 2614186 | -2.057600226 | 3.33E-16 | 7.63E-15 |
| VC0216 | 2614570 | 0.965218224 | 3.85E-08 | 4.02E-07 |
| VC0271 | 2614477 | 1.309574888 | 1.13E-14 | 2.27E-13 |
| VC0272 | 2614478 | 1.158629204 | 0.002255929 | 0.009367367 |
| VC0286 | 2615009 | -1.040937476 | 0.001141309 | 0.005214626 |
| VC0287 | 2615010 | -1.748047379 | 0.000391011 | 0.002003557 |
| VC0288 | 2615011 | -1.112810302 | 0.003667431 | 0.014062579 |
| VC0294 | 2614964 | -1.352939128 | 0.008726196 | 0.029632091 |
| VC0298 | 2614968 | 1.130463365 | 3.91E-07 | 3.56E-06 |
| VC0338 | 2615074 | 1.709499332 | 1.78E-15 | 3.73E-14 |
| VC0354 | 2615067 | 0.995569414 | 3.61E-14 | 6.95E-13 |
| VC0365 | 2615044 | -1.124072788 | 5.56E-09 | 6.54E-08 |
| VC0370 | 2615049 | 0.971707202 | 2.00E-12 | 3.25E-11 |
| VC0475 | 2615269 | -1.845925671 | 8.54E-14 | 1.60E-12 |
| VC0483 | 2615277 | 2.055966581 | 0 | 0 |
| VC0486 | 2615280 | 2.346969029 | 0 | 0 |
| VC0487 | 2615281 | 2.480637179 | 0 | 0 |
| VC0488 | 2615282 | 1.011955522 | 2.35E-07 | 2.18E-06 |
| VC0549 | 2615226 | 1.467268899 | 2.97E-11 | 4.39E-10 |
| VC0566 | 2615243 | 1.001386901 | 0 | 0 |
| VC0589 | 2615377 | 1.424697012 | 3.57E-10 | 4.84E-09 |
| VC0590 | 2615378 | 0.995501706 | 1.07E-05 | 7.82E-05 |
| VC0606 | 2615394 | -2.109132613 | 2.03E-11 | 3.04E-10 |
| VC0629 | 2615417 | -5.388409918 | 0 | 0 |
| VC0676 | 2615465 | 2.119285541 | 0 | 0 |
| VC0711 | 2615715 | 1.652729621 | 2.99E-13 | 5.37E-12 |
| VC0714 | 2615723 | -1.015495475 | 4.04E-08 | 4.20E-07 |
| VC0721 | 2615730 | 0.989646143 | 1.83E-08 | 2.00E-07 |
| VC0737 | 2615746 | 1.581383651 | 2.55E-15 | 5.30E-14 |
| VC0786 | 2615329 | 1.185302991 | 5.87E-09 | 6.88E-08 |
| VC0790 | 2615333 | -1.781410027 | 6.52E-14 | 1.23E-12 |
| VC0791 | 2615334 | -3.080704345 | 0 | 0 |
| VC0792 | 2615335 | -3.750034077 | 0 | 0 |
| VC0794 | 2615337 | -4.00647017 | 0 | 0 |
| VC0795 | 2615338 | -3.850100372 | 0 | 0 |
| VC0796 | 2615339 | -3.87854397 | 0 | 0 |
| VC0797 | 2615340 | -3.279606533 | 0 | 0 |
| VC0798 | 2615341 | -3.563857031 | 0 | 0 |
| VC0799 | 2615342 | -3.516611281 | 0 | 0 |
| VC0800 | 2615343 | -3.302353432 | 0 | 0 |
| VC0801 | 2615344 | -3.450490887 | 0 | 0 |
| VC0819 | 2614486 | -2.534515229 | 0 | 0 |
| VC0820 | 2614487 | -1.577895166 | 2.22E-16 | 5.14E-15 |
| VC0821 | 2614488 | -1.332031056 | 1.56E-11 | 2.37E-10 |
| VC0822 | 2614489 | -1.210785802 | 8.45E-13 | 1.45E-11 |
| VC0823 | 2614490 | -1.127507188 | 3.15E-13 | 5.64E-12 |
| VC0825 | 2614492 | -1.639784031 | 0 | 0 |
| VC0829 | 2614496 | -1.038078051 | 6.26E-10 | 8.12E-09 |
| VC0830 | 2614497 | -1.11271721 | 4.71E-10 | 6.25E-09 |
| VC0831 | 2614498 | -1.116838584 | 7.54E-10 | 9.62E-09 |
| VC0832 | 2614499 | -1.307199586 | 9.30E-14 | 1.73E-12 |
| VC0833 | 2614500 | -1.305143517 | 1.76E-12 | 2.91E-11 |
| VC0834 | 2614501 | -1.214788084 | 4.44E-10 | 5.91E-09 |
| VC0835 | 2614502 | -1.423677704 | 4.84E-13 | 8.54E-12 |
| VC0836 | 2614503 | -1.432341628 | 1.67E-15 | 3.51E-14 |
| VC0845 | 2614512 | -1.388228412 | 1.52E-14 | 3.03E-13 |
| VC0855 | 2614522 | 1.833532548 | 0 | 0 |
| VC0856 | 2614523 | 2.315454882 | 0 | 0 |
| VC0873 | 2614540 | 1.470832076 | 0 | 0 |
| VC0886 | 2614115 | 0.982710519 | 1.05E-05 | 7.74E-05 |
| VC0910 | 2614201 | -2.266638882 | 0 | 0 |
| VC0911 | 2614202 | -2.547583316 | 0 | 0 |
| VC0913 | 2614133 | 1.529160679 | 0 | 0 |
| VC0914 | 2614134 | -12.98208917 | 2.68E-08 | 2.87E-07 |
| VC0916 | 2614136 | 3.613545953 | 0 | 0 |
| VC0917 | 2614137 | 2.390275879 | 2.24E-12 | 3.60E-11 |
| VC0918 | 2614138 | 1.744743446 | 1.21E-11 | 1.85E-10 |
| VC0957 | 2614210 | 1.544898529 | 0 | 0 |
| VC0972 | 2614225 | 1.131181751 | 7.44E-13 | 1.29E-11 |
| VC0977 | 2614230 | 1.789551712 | 1.86E-12 | 3.05E-11 |
| VC1061 | 2614331 | -1.114728041 | 1.21E-05 | 8.74E-05 |
| VC1073 | 2614343 | 1.766310628 | 0 | 0 |
| VC1074 | 2614344 | -1.159814724 | 1.09E-13 | 2.02E-12 |
| VC1080 | 2614350 | 2.108697158 | 0 | 0 |
| VC1081 | 2614351 | 1.435097686 | 3.82E-13 | 6.80E-12 |
| VC1092 | 2614362 | -1.963054246 | 0 | 0 |
| VC1093 | 2614363 | -1.796249993 | 0 | 0 |
| VC1094 | 2614364 | -1.900105899 | 0 | 0 |
| VC1095 | 2614365 | -1.560065324 | 0 | 0 |
| VC1112 | 2614382 | 1.679976074 | 0 | 0 |
| VC1113 | 2614383 | 1.40748046 | 4.72E-12 | 7.42E-11 |
| VC1116 | 2614386 | 4.010684031 | 9.39E-10 | 1.18E-08 |
| VC1117 | 2614387 | 1.305533904 | 0 | 0 |
| VC1119 | 2614389 | -1.110375387 | 0.000121232 | 0.000712282 |
| VC1147 | 2614580 | 1.295613615 | 8.88E-16 | 1.93E-14 |
| VC1153 | 2614586 | 0.985036451 | 1.14E-07 | 1.12E-06 |
| VC1156 | 2614589 | 1.270313657 | 4.92E-10 | 6.49E-09 |
| VC1157 | 2614590 | 1.907997456 | 1.44E-15 | 3.06E-14 |
| VC1217 | 2614654 | 1.224934448 | 1.09E-05 | 7.96E-05 |
| VC1223 | 2614660 | 1.47024748 | 6.91E-12 | 1.07E-10 |
| VC1224 | 2614661 | 1.34441899 | 0 | 0 |
| VC1225 | 2614662 | 1.686311283 | 7.49E-13 | 1.29E-11 |
| VC1264 | 2614718 | -1.92603969 | 0 | 0 |
| VC1265 | 2614719 | -1.813185718 | 0 | 0 |
| VC1266 | 2614720 | -1.726992583 | 0 | 0 |
| VC1267 | 2614721 | -1.514803492 | 0 | 0 |
| VC1269 | 2614723 | 1.540811001 | 0 | 0 |
| VC1272 | 2614726 | -0.985255783 | 8.99E-05 | 0.000549907 |
| VC1298 | 2614752 | 1.180398785 | 9.12E-10 | 1.15E-08 |
| VC1302 | 2614756 | 1.090331556 | 9.92E-09 | 1.13E-07 |
| VC1312 | 2614766 | -1.205515263 | 2.51E-13 | 4.53E-12 |
| VC1316 | 2614770 | -2.445716278 | 0 | 0 |
| VC1317 | 2614771 | -2.379966955 | 0 | 0 |
| VC1318 | 2614772 | -1.992803361 | 0 | 0 |
| VC1319 | 2614773 | -2.279435138 | 0 | 0 |
| VC1320 | 2614774 | -2.107789612 | 0 | 0 |
| VC1329 | 2614783 | -2.60141195 | 0 | 0 |
| VC1343 | 2614797 | 1.50670846 | 0 | 0 |
| VC1358 | 2614812 | 1.208122258 | 5.55E-16 | 1.23E-14 |
| VC1391 | 2614023 | 1.388223596 | 0 | 0 |
| VC1392 | 2614024 | 1.354470925 | 0 | 0 |
| VC1408 | 2614040 | 1.340593139 | 0 | 0 |
| VC1409 | 2614041 | 1.194775733 | 0 | 0 |
| VC1410 | 2614042 | 1.145410771 | 0 | 0 |
| VC1418 | 2614050 | 1.067396576 | 1.11E-16 | 2.60E-15 |
| VC1422 | 2614054 | -1.646881411 | 0 | 0 |
| VC1446 | 2614078 | -1.920604809 | 0 | 0 |
| VC1447 | 2614079 | -2.013689129 | 0 | 0 |
| VC1448 | 2614080 | -2.101873916 | 0 | 0 |
| VC1449 | 2614081 | -2.136710636 | 0 | 0 |
| VC1450 | 2613956 | -1.976130791 | 0 | 0 |
| VC1451 | 2613957 | -1.891691945 | 0 | 0 |
| VC1477 | 2613983 | -0.974564962 | 5.77E-06 | 4.47E-05 |
| VC1510 | 2614016 | 0.964390863 | 1.36E-10 | 1.91E-09 |
| VC1511 | 2614017 | 0.963132565 | 4.44E-16 | 1.00E-14 |
| VC1539 | 2613918 | 1.275173002 | 0 | 0 |
| VC1545 | 2613924 | -1.027682817 | 7.32E-06 | 5.54E-05 |
| VC1548 | 2613927 | -1.105443619 | 4.85E-10 | 6.42E-09 |
| VC1560 | 2613939 | 1.421718364 | 0 | 0 |
| VC1573 | 2613952 | -1.313345575 | 2.11E-12 | 3.41E-11 |
| VC1574 | 2613953 | -1.281145099 | 0 | 0 |
| VC1575 | 2613954 | -1.721748587 | 0 | 0 |
| VC1576 | 2613955 | -1.862786549 | 0 | 0 |
| VC1577 | 2613831 | -1.905055341 | 0 | 0 |
| VC1578 | 2613832 | -1.747642482 | 0 | 0 |
| VC1579 | 2613833 | -1.984068715 | 0 | 0 |
| VC1584 | 2613838 | -1.438628803 | 1.39E-06 | 1.16E-05 |
| VC1607 | 2613863 | 1.12292676 | 8.17E-10 | 1.03E-08 |
| VC1621 | 2613877 | -0.998351728 | 2.11E-15 | 4.40E-14 |
| VC1637 | 2613768 | 1.321808332 | 1.79E-11 | 2.69E-10 |
| VC1638 | 2613769 | 1.323818018 | 5.28E-11 | 7.61E-10 |
| VC1639 | 2613770 | 1.044035084 | 1.11E-16 | 2.60E-15 |
| VC1642 | 2613773 | -1.063422483 | 0.001772214 | 0.007603063 |
| VC1645 | 2613776 | 1.150590198 | 3.00E-08 | 3.20E-07 |
| VC1647 | 2613778 | -1.215374263 | 4.99E-10 | 6.56E-09 |
| VC1649 | 2613780 | -1.901126794 | 0 | 0 |
| VC1651 | 2613782 | 1.022857162 | 0 | 0 |
| VC1652 | 2613783 | 1.262599776 | 0 | 0 |
| VC1653 | 2613784 | 1.206851334 | 3.33E-16 | 7.63E-15 |
| VC1663 | 2613794 | 1.508669489 | 0 | 0 |
| VC1673 | 2613804 | -3.476234526 | 0 | 0 |
| VC1674 | 2613805 | 1.286162641 | 2.22E-16 | 5.14E-15 |
| VC1675 | 2613806 | 1.053569206 | 1.13E-08 | 1.27E-07 |
| VC1688 | 2613819 | -1.282344895 | 0.002015009 | 0.00848124 |
| VC1709 | 2613714 | -2.235586366 | 0 | 0 |
| VC1727 | 2613732 | 1.253997219 | 0 | 0 |
| VC1728 | 2613733 | 1.602221341 | 3.90E-11 | 5.75E-10 |
| VC1744 | 2613749 | 2.306516106 | 0 | 0 |
| VC1745 | 2613750 | 3.031150095 | 0 | 0 |
| VC1746 | 2613751 | 3.819454653 | 0 | 0 |
| VC1748 | 2613753 | 4.858252631 | 0 | 0 |
| VC1749 | 2613754 | 4.73980381 | 0 | 0 |
| VC1750 | 2613755 | 3.785788836 | 0 | 0 |
| VC1756 | 2613761 | 3.461144562 | 0 | 0 |
| VC1757 | 2613762 | -11.32618388 | 0 | 0 |
| VC1761 | 2613767 | -1.10760403 | 5.41E-09 | 6.38E-08 |
| VC1776 | 2613656 | 1.085204616 | 1.69E-12 | 2.79E-11 |
| VC1779 | 2613659 | 1.496646888 | 1.11E-15 | 2.39E-14 |
| VC1781 | 2613661 | 1.137225231 | 1.71E-11 | 2.57E-10 |
| VC1784 | 2613664 | 1.202348639 | 1.38E-12 | 2.33E-11 |
| VC1820 | 2613700 | 2.120515971 | 0 | 0 |
| VC1821 | 2613701 | 2.543977728 | 0 | 0 |
| VC1825 | 2613579 | 1.492610626 | 0 | 0 |
| VC1826 | 2613580 | 2.94742719 | 0 | 0 |
| VC1827 | 2613581 | 3.090192019 | 0 | 0 |
| VC1828 | 2613582 | 2.629930093 | 0 | 0 |
| VC1829 | 2613583 | 2.20858494 | 5.55E-08 | 5.66E-07 |
| VC1830 | 2613584 | 2.113462659 | 0.000902204 | 0.004263128 |
| VC1831 | 2613585 | 1.21110116 | 1.11E-15 | 2.39E-14 |
| VC1953 | 2613457 | -1.306566863 | 1.72E-08 | 1.89E-07 |
| VC1964 | 2613468 | 1.687366104 | 0 | 0 |
| VC1991 | 2613495 | 2.236482345 | 0 | 0 |
| VC2070 | 2613450 | 1.305297799 | 2.14E-11 | 3.18E-10 |
| VC2076 | 2613332 | -1.117555348 | 3.47E-08 | 3.65E-07 |
| VC2105 | 2613361 | 1.048698648 | 0.001135292 | 0.005193052 |
| VC2212 | 2613251 | 1.658374895 | 0 | 0 |
| VC2305 | 2613101 | 1.300429334 | 0 | 0 |
| VC2323 | 2613119 | -1.052111101 | 6.73E-09 | 7.79E-08 |
| VC2356 | 2613152 | -1.030484718 | 0 | 0 |
| VC2357 | 2613153 | 1.039583874 | 0.000511096 | 0.002550448 |
| VC2362 | 2613031 | 1.477633476 | 0 | 0 |
| VC2363 | 2613032 | 1.730261623 | 0 | 0 |
| VC2364 | 2613033 | 1.488435792 | 0 | 0 |
| VC2388 | 2613057 | -1.203473278 | 1.75E-08 | 1.92E-07 |
| VC2436 | 2612978 | 2.192826625 | 0 | 0 |
| VC2473 | 2613015 | 1.542702815 | 4.44E-16 | 1.00E-14 |
| VC2485 | 2615142 | 2.530722309 | 0 | 0 |
| VC2496 | 2615159 | -1.070884031 | 2.00E-09 | 2.43E-08 |
| VC2565 | 2615582 | 1.099564409 | 1.59E-14 | 3.15E-13 |
| VC2569 | 2615586 | -1.424218238 | 6.66E-16 | 1.46E-14 |
| VC2642 | 2615659 | 1.023654546 | 3.68E-08 | 3.85E-07 |
| VC2656 | 2615673 | 1.018693409 | 4.27E-11 | 6.25E-10 |
| VC2657 | 2615674 | 0.985662093 | 1.48E-11 | 2.26E-10 |
| VC2658 | 2615675 | 0.992334332 | 9.98E-13 | 1.70E-11 |
| VC2662 | 2615679 | 1.694770528 | 0 | 0 |
| VC2664 | 2615492 | 1.854109851 | 0 | 0 |
| VC2665 | 2615493 | 1.964567563 | 0 | 0 |
| VC2674 | 2615502 | 1.463988688 | 1.38E-12 | 2.33E-11 |
| VC2675 | 2615503 | 1.694665303 | 2.00E-14 | 3.89E-13 |
| VC2691 | 2615519 | 3.810574078 | 0 | 0 |
| VC2692 | 2615520 | 1.739796319 | 0 | 0 |
| VC2693 | 2615521 | 1.566596414 | 0 | 0 |
| VC2694 | 2615522 | 1.34673212 | 1.08E-11 | 1.66E-10 |
| VC2698 | 2615526 | 1.882433665 | 0 | 0 |
| VC2699 | 2615527 | 1.18932985 | 0 | 0 |
| VC2706 | 2615534 | -1.986751692 | 0 | 0 |
| VC2714 | 2615542 | 1.068189512 | 0 | 0 |
| VC2738 | 2614901 | 1.5030191 | 0 | 0 |
| VC2753 | 2614930 | -1.139375428 | 4.27E-10 | 5.74E-09 |
| VCA0013 | 2612304 | -1.823266392 | 0 | 0 |
| VCA0014 | 2612495 | -1.792202986 | 0 | 0 |
| VCA0025 | 2612526 | 1.027772892 | 5.08E-08 | 5.23E-07 |
| VCA0029 | 2612470 | -1.147727012 | 4.83E-11 | 7.01E-10 |
| VCA0030 | 2612479 | -1.155793085 | 4.77E-15 | 9.81E-14 |
| VCA0035 | 2612439 | 2.123245831 | 0 | 0 |
| VCA0070 | 2612095 | 2.060988753 | 6.78E-13 | 1.18E-11 |
| VCA0071 | 2612071 | 3.122095704 | 6.67E-10 | 8.57E-09 |
| VCA0072 | 2612084 | 2.247976386 | 6.48E-09 | 7.53E-08 |
| VCA0073 | 2612079 | 1.892983087 | 5.61E-12 | 8.74E-11 |
| VCA0078 | 2612060 | 1.156230138 | 3.06E-07 | 2.80E-06 |
| VCA0083 | 2612023 | 1.859987986 | 0 | 0 |
| VCA0125 | 2612541 | 1.149667464 | 0 | 0 |
| VCA0128 | 2612792 | 1.087003156 | 0 | 0 |
| VCA0129 | 2612536 | 1.768997772 | 0 | 0 |
| VCA0130 | 2612537 | 2.464578342 | 0 | 0 |
| VCA0131 | 2612762 | 2.404578201 | 0 | 0 |
| VCA0132 | 2612711 | 1.124806268 | 0 | 0 |
| VCA0139 | 2612665 | 3.065910038 | 0 | 0 |
| VCA0148 | 2612881 | 1.465171977 | 1.22E-15 | 2.60E-14 |
| VCA0151 | 2612539 | 1.749042399 | 0 | 0 |
| VCA0152 | 2612756 | 1.68637696 | 1.14E-05 | 8.29E-05 |
| VCA0159 | 2612390 | 1.897112485 | 2.34E-13 | 4.24E-12 |
| VCA0165 | 2611905 | 1.021496176 | 3.95E-12 | 6.27E-11 |
| VCA0217 | 2612625 | -1.655610087 | 6.83E-08 | 6.86E-07 |
| VCA0227 | 2612388 | -2.409471243 | 0 | 0 |
| VCA0228 | 2612389 | -2.008006778 | 6.66E-16 | 1.46E-14 |
| VCA0229 | 2612771 | -2.38249695 | 0 | 0 |
| VCA0230 | 2612477 | -2.313694479 | 0 | 0 |
| VCA0231 | 2612478 | -1.09785056 | 1.61E-06 | 1.32E-05 |
| VCA0241 | 2612429 | 1.218715592 | 0.000721395 | 0.003458434 |
| VCA0242 | 2612452 | 1.137104703 | 0.002457018 | 0.010015533 |
| VCA0249 | 2612200 | 1.832807014 | 0 | 0 |
| VCA0259 | 2612122 | -1.65307448 | 0.001144862 | 0.005224898 |
| VCA0262 | 2612130 | -0.976180949 | 0.000816148 | 0.00388859 |
| VCA0270 | 2612075 | 1.346761908 | 0 | 0 |
| VCA0271 | 2612066 | 1.058331607 | 2.51E-10 | 3.45E-09 |
| VCA0279 | 2612059 | 1.395056695 | 9.72E-13 | 1.66E-11 |
| VCA0294 | 2611955 | 1.006929719 | 0.00111287 | 0.00511384 |
| VCA0325 | 2612766 | -1.641064784 | 0.001774738 | 0.00760575 |
| VCA0416 | 2612094 | -1.545606696 | 0.00032665 | 0.0017065 |
| VCA0420 | 2612070 | -1.030297969 | 0.000408847 | 0.002084285 |
| VCA0427 | 2612021 | -1.15230493 | 0.001975385 | 0.008340747 |
| VCA0429 | 2612014 | -1.737130727 | 0.001902334 | 0.008057773 |
| VCA0431 | 2611998 | -1.524908905 | 0.000363654 | 0.001870553 |
| VCA0446 | 2611941 | 0.975366876 | 2.45E-05 | 0.0001676 |
| VCA0447 | 2611936 | 1.079626094 | 1.82E-07 | 1.72E-06 |
| VCA0448 | 2611937 | 1.091371575 | 0.001588069 | 0.006954527 |
| VCA0454 | 2611915 | -1.108386661 | 1.43E-06 | 1.19E-05 |
| VCA0464 | 2611877 | -1.153133999 | 0.000147602 | 0.000849774 |
| VCA0467 | 2611853 | -1.715184648 | 0.008688308 | 0.029565209 |
| VCA0473 | 2612339 | -1.160208927 | 0.002635674 | 0.010635596 |
| VCA0493 | 2612932 | -1.039903806 | 6.25E-07 | 5.47E-06 |
| VCA0496 | 2612923 | -1.164819872 | 1.36E-07 | 1.30E-06 |
| VCA0514 | 2612562 | 1.191962787 | 0 | 0 |
| VCA0515 | 2612386 | 0.963918875 | 0.010234515 | 0.033920348 |
| VCA0516 | 2612387 | -1.260337746 | 0.001853457 | 0.007867373 |
| VCA0517 | 2612820 | -1.530862161 | 0.000487904 | 0.002452987 |
| VCA0518 | 2612791 | -1.602784036 | 0.000678598 | 0.003280028 |
| VCA0519 | 2612773 | -1.059747455 | 0.017657806 | 0.053928986 |
| VCA0538 | 2612258 | 1.731519003 | 0 | 0 |
| VCA0539 | 2612254 | 1.954110825 | 0 | 0 |
| VCA0540 | 2612255 | -1.573659571 | 0 | 0 |
| VCA0556 | 2612312 | 1.83840612 | 0 | 0 |
| VCA0576 | 2612698 | -1.084165245 | 1.50E-08 | 1.66E-07 |
| VCA0594 | 2612811 | 1.740765897 | 0 | 0 |
| VCA0625 | 2612781 | -0.984576244 | 6.44E-10 | 8.32E-09 |
| VCA0638 | 2612936 | -4.956012912 | 4.15E-12 | 6.58E-11 |
| VCA0639 | 2612937 | 1.080565282 | 0.01046818 | 0.034580377 |
| VCA0640 | 2612743 | 1.275137341 | 0.00791621 | 0.027312931 |
| VCA0641 | 2612744 | 2.574358157 | 1.23E-05 | 8.87E-05 |
| VCA0644 | 2612658 | 1.299224946 | 6.97E-11 | 9.98E-10 |
| VCA0657 | 2612772 | 1.265955615 | 1.28E-07 | 1.23E-06 |
| VCA0665 | 2612512 | -1.061374664 | 4.12E-11 | 6.04E-10 |
| VCA0702 | 2612085 | 1.664382293 | 0 | 0 |
| VCA0720 | 2611992 | -1.032519236 | 0.011099392 | 0.036336 |
| VCA0721 | 2611985 | 2.739223163 | 0 | 0 |
| VCA0722 | 2611986 | 1.666391841 | 0 | 0 |
| VCA0732 | 2611948 | 2.796183039 | 0 | 0 |
| VCA0747 | 2611906 | 1.464081017 | 1.60E-10 | 2.25E-09 |
| VCA0752 | 2611893 | 1.197719921 | 7.34E-06 | 5.55E-05 |
| VCA0754 | 2611874 | -1.145936204 | 3.30E-08 | 3.48E-07 |
| VCA0760 | 2611827 | 1.026156204 | 0.000352844 | 0.001821968 |
| VCA0780 | 2612673 | 1.122405529 | 1.35E-08 | 1.51E-07 |
| VCA0781 | 2612660 | 1.544328571 | 0 | 0 |
| VCA0782 | 2612661 | 1.731630146 | 0 | 0 |
| VCA0783 | 2612644 | 1.054315748 | 1.22E-15 | 2.60E-14 |
| VCA0798 | 2611825 | 1.458673091 | 7.80E-14 | 1.47E-12 |
| VCA0802 | 2612775 | -1.615499036 | 0 | 0 |
| VCA0803 | 2612776 | -1.041999771 | 0.000634822 | 0.003098333 |
| VCA0808 | 2611881 | 1.017408146 | 0 | 0 |
| VCA0810 | 2611872 | 2.873887222 | 1.48E-05 | 0.000105422 |
| VCA0811 | 2611865 | 2.130547373 | 0 | 0 |
| VCA0845 | 2612245 | 2.257114012 | 0 | 0 |
| VCA0846 | 2612234 | 1.883673825 | 0 | 0 |
| VCA0847 | 2612217 | 1.065310426 | 8.13E-10 | 1.03E-08 |
| VCA0860 | 2612900 | -1.618350363 | 0 | 0 |
| VCA0862 | 2612849 | -2.940011505 | 0 | 0 |
| VCA0863 | 2612850 | -2.841168989 | 0 | 0 |
| VCA0867 | 2612873 | 1.227312354 | 1.15E-11 | 1.77E-10 |
| VCA0868 | 2612871 | -1.297070326 | 0.012427065 | 0.039932037 |
| VCA0874 | 2612585 | -1.697501685 | 1.03E-07 | 1.02E-06 |
| VCA0877 | 2612558 | 1.059968657 | 2.90E-09 | 3.48E-08 |
| VCA0909 | 2612225 | -1.825179651 | 0 | 0 |
| VCA0910 | 2612226 | -1.461853901 | 1.22E-06 | 1.03E-05 |
| VCA0911 | 2612220 | -1.476993453 | 6.13E-07 | 5.37E-06 |
| VCA0912 | 2612211 | -1.466666652 | 0.000151503 | 0.000867756 |
| VCA0913 | 2612212 | -1.552761918 | 2.96E-07 | 2.72E-06 |
| VCA0914 | 2612931 | -1.169437009 | 3.66E-06 | 2.89E-05 |
| VCA0933 | 2612854 | -2.161561824 | 0 | 0 |
| VCA0934 | 2612855 | -1.859147179 | 7.71E-10 | 9.80E-09 |
| VCA0935 | 2612835 | -2.653587805 | 1.55E-08 | 1.71E-07 |
| VCA0943 | 2612568 | -1.061335017 | 3.48E-10 | 4.74E-09 |
| VCA0944 | 2612560 | -1.54433603 | 0 | 0 |
| VCA0945 | 2612561 | -1.127183209 | 0 | 0 |
| VCA0946 | 2612547 | -1.431283193 | 0 | 0 |
| VCA0962 | 2612393 | 1.880285152 | 0 | 0 |
| VCA0966 | 2612718 | 0.988333805 | 0.00234115 | 0.009651957 |
| VCA0983 | 2612816 | 1.06130154 | 4.85E-07 | 4.35E-06 |
| VCA0985 | 2612794 | 1.11559166 | 0 | 0 |
| VCA0986 | 2612795 | 1.076888115 | 1.05E-06 | 8.95E-06 |
| VCA0987 | 2612344 | 1.060960231 | 3.12E-14 | 6.04E-13 |
| VCA0992 | 2612305 | 1.217994447 | 0.003742471 | 0.014281982 |
| VCA0998 | 2612248 | 1.444447145 | 0 | 0 |
| VCA1006 | 2612546 | 1.283147051 | 0.014726087 | 0.046280337 |
| VCA1027 | 2612419 | -0.988851208 | 1.70E-09 | 2.08E-08 |
| VCA1028 | 2612207 | -2.978059082 | 0 | 0 |
| VCA1051 | 2612097 | 1.350300476 | 2.26E-12 | 3.63E-11 |
| VCA1069 | 2612009 | 1.084918111 | 2.63E-10 | 3.61E-09 |
| VCA1071 | 2612001 | -1.239316692 | 6.99E-15 | 1.41E-13 |
| VCA1072 | 2611994 | -1.04235645 | 1.11E-16 | 2.60E-15 |
| VCA1106 | 2612780 | 1.268370431 | 0.001198451 | 0.005444662 |
| VCA1107 | 2612414 | 1.168354434 | 0.009404987 | 0.031483529 |
