# Supplementary material for: The Vibrio cholerae RND efflux systems impact virulence factor production and adaptive responses via periplasmic sensor proteins
Source: PLoS Pathog. 2018 Jan 5;14(1):e1006804. doi: 10.1371/journal.ppat.1006804 (PMC5773229; doi:10.1371/journal.ppat.1006804)
Supplement: S2 Table — (DOCX) [file ppat.1006804.s008.docx]

**TABLE S4. Oligonucleotide primers used in this study.**

| **Primers:** | | **Sequence: 5’ 🡪 3’:** | |
| --- | --- | --- | --- |
|  | **Cloning primers** |  | |
|  | P*_ctxAB_*-F | AACTCGAGAAGCGCTGTGGGTAGAAGTG | |
|  | P*_ctxAB_*-R | CCTCTAGAACACAAATATTATCTTTACC | |
|  | P*_tcpA_*-F | TCCTCGAGTGCCAGATCCTAGCTCTCAG | |
|  | P*_tcpA_*-R | GGTCTAGATTACAAACTTCTTCTTAAAAAGCTG | |
|  | *aphB*-F | GATCGGAATTCATAAATTAGCGATAGTTGCAACATAATGTG | |
|  | *aphB*-R | GGTCTAGAAAAGGGCGCGAAGCCCTTTTTCTATTG | |
|  | P*_rtxB_*-F | CCTCTAGATCGCCGATGTATTACAGC | |
|  | PrtxB-R | CCCTCGAGCTGTCTGAACGAACTTCTC | |
|  | Vc.*lacZ*.F.BamHI | AAGGATCCAGCGGTGAGCTTGCAGCCAAGAG | |
|  | Vc.*lacZ*.R.SacI | GGGAGCTCGGCAACCATAAAAGACGTAGCACGTGAAG | |
|  | **qRT-PCR primers** | |  |
|  | *leuO*-F | GACCACTTCGCCACAAATCACCA | |
|  | *leuO*-R | CGTTGGATGGCGGAAAATGCG | |
|  | *aphA*-F | GCAGAACCTTACCGTCTGCAA | |
|  | *aphA*-R | GCGTAATAAGCGGCTTCGATT | |
|  | *aphB*-F | ATCGGTGAAGTGAAAGACATTTTG | |
|  | *aphB*-R | GATGTTGATGCAACTCTTCAGCAT | |
|  | *tcpP*-F | GGTGGAGTTATGGCCAATGG | |
|  | *tcpP*-R | GTTATCCCCGGTAACCTTGCT | |
|  | *toxR*-F | GTCAAAACGGTTCCGAAACG | |
|  | *toxR*-R | TGTCATGAGCAGCTTCGCTTT | |
|  | *gyrA*-F | CAATGCCGGTACACTGGTACG | |
|  | *gyrA*-R | AAGTACGGATCAGGGTCACG | |
